# Oncolytic virus M1 reinvigorates CD8^+^ T-cell immunity against glioblastoma through B-cell-dependent antigen cross-presentation in the spleen

**DOI:** 10.1038/s41423-026-01396-w

**Published:** 2026-03-04

**Authors:** Yu Han, Cui Guo, Chaoxin Chen, Wenzhuo Yang, Honghui Li, Changjia He, Jialin Deng, Zhijie Chen, Wenfeng Liu, Jiehong Chen, Yingqian Zhong, Caixin Yan, Cuiying Xu, Xuanming Liang, Sheng Zhong, Furong Chen, Depei Li, Cong Li, Wanming Hu, Zhenghe Chen, Yonggao Mou, Zhongping Chen, Guangmei Yan, Michael Lim, Wenbo Zhu, Ke Sai

**Affiliations:** 1 Department of Neurosurgery, State Key Laboratory of Oncology in South China, Guangdong Provincial Clinical Research Center for Cancer, Sun Yat-sen University Cancer Center, Guangzhou, China; 2 Department of Pharmacology, Zhongshan School of Medicine, Sun Yat-sen University, Guangzhou, China; 3 Department of Neurosurgery, The Third Affiliated Hospital of Sun Yat-sen University Lingnan Hospital, Guangzhou, China; 4 The Second Affiliated Hospital of Guangzhou University of Chinese Medicine, Guangdong Provincial Hospital of Chinese Medicine, Guangzhou, China; 5 Department of Pathology, State Key Laboratory of Oncology in South China, Guangdong Provincial Clinical Research Center for Cancer, Sun Yat-sen University Cancer Center, Guangzhou, China; 6 Department of Neurosurgery, Stanford School of Medicine, Palo Alto, CA USA

**Keywords:** Glioma, Oncolytic virus, B cell, Antigen cross-presentation, CNS cancer, Tumour immunology

## Abstract

Glioblastoma multiforme (GBM) is a lethal primary brain cancer with limited treatment options. Systemic and local immunosuppression induced by GBMs contributes to malignancy aggressiveness and resistance to immune checkpoint blockade (ICB) therapy. Herein, we demonstrated that a novel oncolytic virus, M1 (OVM), reversed GBM-driven systemic immunosuppression and promoted T lymphocyte infiltration within the tumor microenvironment (TME). Intravenous administration of OVM suppressed glioma progression in a spleen-dependent manner. Mechanistically, OVM enhanced B-cell–T-cell interactions in the spleen through the formation of immune synapses. A subset of B cells positive for bone marrow stromal cell antigen 2 (Bst2) was enriched in the splenic marginal zone following OVM treatment and exhibited superior capacity for antigen cross-presentation. These splenic Bst2^+^ B cells activated cognate CD8^+^ T cells to mediate adaptive antitumor immunity against intracranial gliomas. Moreover, OVM treatment synergized with anti-PD-1 therapy and further extended the survival of glioma-bearing animals. Collectively, our findings highlight the therapeutic potential of intravenous OVM for GBM management and reveal a novel immunomodulatory mechanism underlying oncolytic virotherapy.

## Introduction

Glioblastoma multiforme (GBM) is the most common and lethal primary brain tumor in adults. Despite aggressive multidisciplinary treatments, the prognosis of GBM remains dismal, with a median overall survival of less than 2 years [1]. Although immune checkpoint blockade (ICB)-based therapy has revolutionized the standard care of many solid tumors, GBMs exhibit profound resistance to such interventions [2]. The systemic depletion of lymphocytes, deficit in antigen presentation, and scarcity of tumor-infiltrating lymphocytes (TILs) contribute to the immunological coldness of fatal malignancies [3, 4]. Innovative strategies to overcome systemic and local immunosuppression are urgently needed to restore immune surveillance in GBM patients.

Oncolytic virotherapy represents an attractive therapeutic approach for gliomas. Emerging evidence indicates that locoregional administration of oncolytic viruses (OVs) is well tolerated and confers durable clinical benefit in a subset of GBM patients [5–7]. Recently, alphavirus M1 (OVM), a novel intravenously administered oncolytic virus, has been explored for glioma treatment. OVM is a strain of Getah-like alphavirus originally isolated from Culex mosquitoes that selectively replicates in tumor cells overexpressing matrix remodeling-associated 8 (MXRA8) [8]. Repeated intravenous administration of OVM is nonpathogenic for nonhuman primates and shows acceptable toxicity with promising efficacy in hepatocellular carcinoma (HCC) patients [9, 10]. Preliminary data have demonstrated that intravenously delivered OVM efficiently penetrates the blood‒brain barrier (BBB) and exhibits antiglioma activity in preclinical models [11]. On the basis of their safety profiles and potential therapeutic benefits, the U.S. Food and Drug Administration (FDA) granted an OVM orphan drug designation (ODD) for malignant gliomas in 2022 [12]. A clinical study (NCT07093814) evaluating the safety and preliminary efficacy of OVM in recurrent glioblastoma is ongoing. However, the mechanism by which OVM initiates adaptive immunity against gliomas is still unclear. Moreover, intravenous OVM treatment triggers a robust systemic antiviral response. Knowledge regarding the impact of the interplay between OVM and the host immune system on therapeutic effects also requires addressing.

In the present study, we investigated the antiglioma activity of OVM and characterized the immune cellular composition of the TME and extratumoral immune compartments. We demonstrated that intravenous delivery of OVM increased the survival of immunocompetent GBM-bearing mice. Comprehensive immunoprofiling revealed that OVM mitigated glioma-driven systemic lymphopenia and promoted CD8^+^ T-cell infiltration in the TME. Notably, we identified the critical role of the spleen and B cells in mediating the antitumor effects of OVM. A subpopulation of splenic B cells positive for bone marrow stromal cell antigen 2 (Bst2) was enriched following OVM treatment and subjected to antigen cross-presentation to activate CD8^+^ T cells for tumor-specific immune responses. Moreover, OVM synergized with a PD-1 inhibitor to significantly suppress the growth of intracranial gliomas.

## Results

### OVM exerts antiglioma activity in vitro

**Fig. 1:**
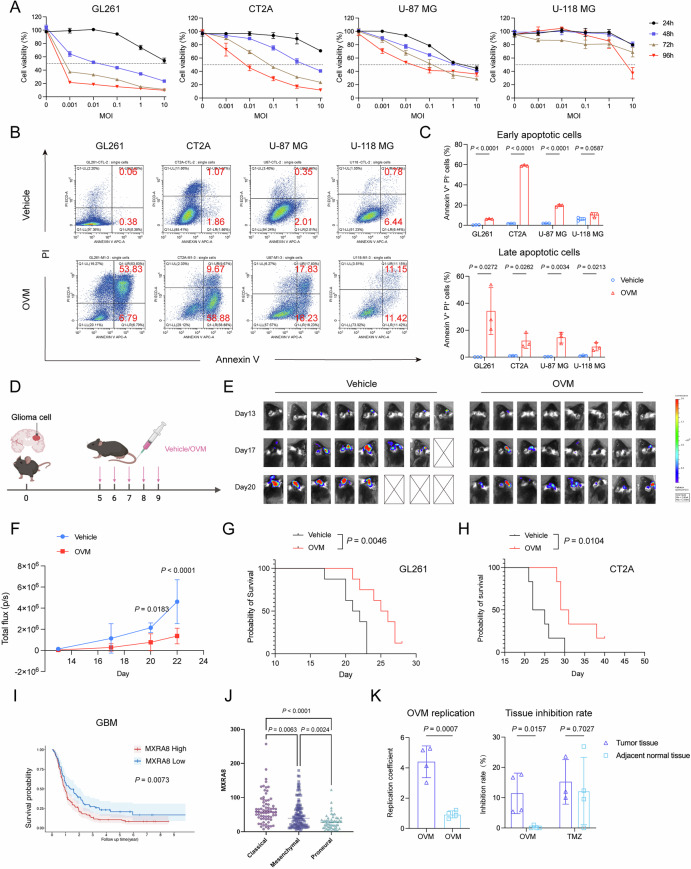
OVM exhibits antiglioma activity. **A** Glioma cell lines GL261, CT2A, U-87 MG, and U-118 MG were infected with OVM at MOIs of 0.001, 0.01, 0.1, 1, and 10, respectively. Cell viability was assessed via the MTT assay at 24, 48, 72, and 96 h postinfection. **B** Glioma apoptosis was evaluated by Annexin V/PI staining following infection with OVM at an MOI of 1 for 48 h. **C** Quantification of early apoptotic cells (Annexin V^+^ PI^−^) and late apoptotic cells (Annexin V^+^ PI^+^). **D** Schematic of the in vivo treatment schedule. Glioma cells (GL261, CT2A, or GL261-Luc) were intracranially implanted on Day 0, followed by daily tail vein injections of vehicle or OVM from Day 5 to Day 9. **E** Bioluminescence imaging of GL261-Luc tumors was performed via the IVIS Spectrum system on days 13, 17, and 20. **F** Tumor burden was quantified by total flux (photons/second). OS of GL261 (**G**) and CT2A (**H**) orthotopic tumor-bearing mice treated with OVM was assessed and represented by Kaplan‒Meier survival curves. **I** Kaplan‒Meier survival curves of GBM patients from the CGGA database stratified by MXRA8 expression. **J** Analysis of MXRA8 expression across classical, mesenchymal and proneural GBM subtypes in the CGGA database. **K** Human glioma tissue fragments (~1 mm³) were treated with OVM (150 µL, 1.16 × 10^8^ CCID_50_/mL), vehicle, or TMZ (50 mg/mL) for 72 h. Viral replication was quantified via qPCR, and replication coefficients were calculated as the ratio of viral copies in standard culture to those in parallel inactivated controls; a coefficient >2 indicated productive viral replication. Tissue viability was assessed by MTT staining

OVM has been shown to multiply and effectively repress growth in various cancer types, including hepatocellular carcinoma, breast cancer and melanoma [13–15]. To determine the antiglioma activity of OVM, we infected 4 widely used murine and human GBM cell lines (GL261, CT2A, U-87 MG, and U-118 MG) with GFP-expressing OVM. After 24 h of infection, strong green fluorescence was detected in all 4 cell lines (Supplementary Fig. S1A), indicating tumor permissivity to the virus. Moreover, OVM exhibited significant cytotoxicity against glioma cells. A greater than 50% reduction in cell viability was observed in 3 out of 4 cell lines after 96 h of exposure to OVM at a multiplicity of infection (MOI) of 0.1 or less (Fig. 1A). In line with previous reports, we found that OVM induced significant apoptotic death in glioma cells (Fig. 1B). Quantitative analysis via flow cytometry revealed that incubation with OVM at an MOI of 1 for 48 h resulted in a 3- to 135-fold increase in the number of Annexin V-positive cells in all 4 cell lines (Fig. 1C).

### Intravenous administration of OVM represses glioma growth in murine glioma models

In an initial experiment to investigate the therapeutic potential of OVM, we treated immunocompromised mice with intravenous injection of OVM (300 µL, titer: 2 × 10^7^ 50% cell culture infectious dose/mL (CCID_50_/mL)) via subcutaneous U87 MG tumors for 5 consecutive days. The results demonstrated that intravenous OVM delivery led to a decrease in the volume and weight of subcutaneous gliomas (Supplementary Fig. S1B, C), which indicated tumor tropism and direct cytotoxicity of OVM. We then asked whether OVM could efficiently cross the BBB and exert antiglioma effects in immunocompetent animals. To this end, we first constructed a reporter virus that encodes a near-infrared fluorescent protein (OVM-iRFP) to track the distribution of OVM in an orthotopic GL261 mouse model. As shown in Supplementary Fig. S1D, fluorescence signals aggregated intracranially in OVM-treated mice but not in control mice. Second, viral RNA copies were quantified in intracranial tumors and adjacent normal brain tissues following intravenous treatment with OVM. Viral RNA was enriched significantly in tumor specimens, whereas that in adjacent normal brain tissues was negligible (Supplementary Fig. S1E). Furthermore, we intracranially injected mice with 2 syngeneic GBM cell lines (GL261 and CT2A) and treated them with intravenous OVM (Fig. 1D). We found that OVM significantly inhibited the growth of glioma and prolonged the survival of glioma-bearing mice (Fig. 1E–H).

### OVM preferentially replicates in and is toxic to human glioma tissues

We previously identified MXRA8 as the entry receptor of OVM, which is upregulated in human glioma compared with corresponding normal tissues [8]. To further determine the clinical significance of MXRA8 expression, we compared MXRA8 expression across different WHO grades in the Chinese Glioma Genome Atlas (CGGA) database. We found that increased MXRA8 expression was positively correlated with WHO tumor grade (Supplementary Fig. S1F) and indicated an unfavorable prognosis in glioma patients (Supplementary Fig. S1G). Similarly, upregulated MXRA8 expression was associated with shorter survival in GBM patients (Fig. 1I). Intertumoral heterogeneity is a hallmark of GBM. Three subtypes (proneural, classical and mesenchymal) with distinct molecular features and clinical outcomes have been classified [16]. We found that MXRA8 expression was significantly elevated in the classical and mesenchymal subtypes (Fig. 1J). These findings suggest that gliomas, especially those with aggressive phenotypes, have increased expression of OVM entry receptors and are more likely to be targeted by the virus. In an effort for clinical translation, we obtained freshly resected GBM samples and paired tumor-adjacent normal tissues from four patients to examine the antitumor effects of OVM. Our results demonstrated that OVM selectively replicated in and caused significant damage to GBM tissues but not to paired nontumor tissues (Fig. 1K). In comparison, temozolomide (TMZ), the standard of care for GBMs, has a prominent inhibitory effect on glioma tissues, whereas it is also toxic to normal tissues (Fig. 1K). In summary, we demonstrated that OVM is a glioma-targeting agent and has potential for clinical translation.

### OVM induces immunogenic cell death and promotes the infiltration of T lymphocytes in the TME

Immunogenic cell death (ICD) is a regulated form of cellular death characterized by the spatiotemporal release of damage-associated molecular patterns (DAMPs) that activate adaptive immunity [17]. To determine the immunogenicity of OVM-triggered apoptotic death, we examined the release of DAMPs, including externalized calreticulin (CRT) and adenosine triphosphate (ATP), in glioma cells. Flow cytometry analysis demonstrated that OVM elicited significant CRT exposure in GL261 and CT2A cells (Supplementary Fig. S2A). Elevated extracellular ATP was also identified in the supernatant collected from both cell lines upon OVM infection (Supplementary Fig. S2B).

**Fig. 2:**
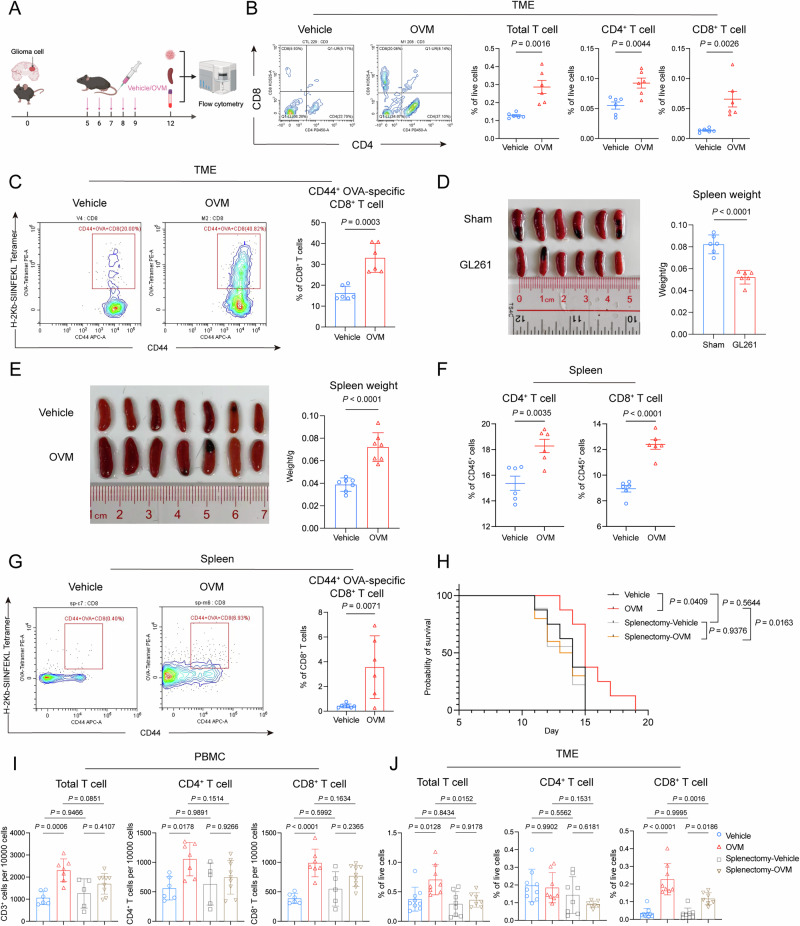
OVM reverses GBM-induced systemic immunosuppression, and the spleen is crucial for the therapeutic efficacy of OVM. **A** GL261 or GL261-OVA glioma cells were intracranially implanted on Day 0. The mice were treated with daily tail vein injections of OVM from days 5 to 9, and the tumors, spleens, and PBMCs were collected on day 12 for flow cytometry analysis. **B** Flow cytometric analysis of T-cell populations within the TME. CD3^+^ total, CD4^+^, and CD8^+^ T cells were quantified. **C** H-2Kb-SIINFEKL tetramer analysis of OVA-specific CD44^+^ CD8^+^ T cells in the TME. **D**, **E** Histology and weight of the spleen. **F**, **G** Flow cytometric analysis of splenic T cells. The proportions of CD4^+^ and CD8^+^ T cells (**F**), as well as OVA-specific CD44^+^ CD8^+^ T cells (**G**), were quantified in GL261 tumor-bearing mice treated with vehicle or OVM. **H** Kaplan‒Meier survival curves of GL261 glioma-bearing mice treated with vehicle or OVM, with or without splenectomy. **I**, **J** Counts of T-cell subtypes were quantified in PBMCs, and their proportions in the TME were quantified

Next, we profiled the immune infiltrate to elucidate the immunomodulatory effects of OVM on the TME. We intracranially injected GL261 cells into syngeneic C57BL/6 mice and systemically administered 5 doses of OVM (300 µL, 2 × 10^7^ CCID_50_/mL) after the establishment of orthotopic tumors (Fig. 2A). Glioma tissues were obtained for immune profiling on day 12. Quantitative analysis revealed that OVM enhanced the infiltration of total CD3^+^ T cells and subsets (CD4^+^ and CD8^+^ T lymphocytes) into the TME (Fig. 2B), whereas no significant changes were observed in other immune cell subpopulations, such as natural killer (NK) cells, B cells, dendritic cells (DCs) or macrophages (Supplementary Fig. S2C, D), following OVM treatment. Although OVM augments T-cell infiltration, the tumor specificity of these T lymphocytes remains to be clarified. To this end, GL261 glioma cells stably expressing full-length ovalbumin (OVA) were orthotopically implanted into syngeneic mice, and H-2Kb-SIINFEKL tetramers were used to track tumor-specific CD8^+^ T cells. We found that the frequency of H-2Kb-SIINFEKL^+^ CD8^+^ T cells significantly increased in the TME following OVM treatment (Fig. 2C). Overall, these findings indicate that intravenous OVM administration reshaped the immunologically cold glioma microenvironment by recruiting tumor-specific CD8^+^ T lymphocytes.

### OVM contracts GBM-induced systemic immunosuppression

GBM patients frequently develop profound systemic immunosuppression, manifesting as lymphopenia and spleen involution [18]. Herein, we recapitulated these changes in murine glioma. We found dramatically diminished frequencies of peripheral CD4^+^ and CD8^+^ T-cell compartments after intracranial implantation of GL261 cells (Supplementary Fig. S2E). An overall reduction in spleen size and weight was also noted (Fig. 2D), accompanied by a significant decrease in the splenic T-cell count (Supplementary Fig. S2F). Histological examination and immunostaining revealed structural disorganization of the spleen characterized by hypoplasia of the white pulp and elevated apoptotic indices (Supplementary Fig. S2G, H). OVM has been reported to reprogram the immunologically shielded TME toward a proinflammatory state [19], whereas its impact on systemic immunity is still unclear. In the present study, we found that the number of CD4^+^ and CD8^+^ T cells increased significantly in the peripheral blood of glioma-bearing mice following intravenous OVM treatment (Supplementary Fig. S2I). Spleen atrophy and loss of the splenic T-cell population were also reversed (Fig. 2E, F). Moreover, we investigated the levels of immunosuppressive cytokines in the peripheral blood of glioma-bearing mice following intravenous OVM treatment. We found that OVM significantly reduced the serum IL-10 and TGF-β concentrations (Supplementary Fig. 2J). These findings indicate that OVM promotes the restoration of systemic immunity. We next sought to determine whether OVM-induced reversal of systemic immunosuppression could enhance tumor-specific immune responses. We isolated splenocytes from GL261-OVA-bearing mice and assessed the presence of tumor-specific CD8^+^ T cells in the spleen by using H-2Kb-SIINFEKL tetramers. Quantification revealed that the frequency of OVA-specific CD8^+^ T cells increased significantly from 0.5% to 4% in the spleens of glioma-bearing mice following OVM treatment (Fig. 2G). Collectively, these results indicate that intravenous OVM reversed GBM-driven systemic immunosuppression and led to the activation of tumor-specific immunity.

### Splenectomy abrogates the antiglioma activity of OVM

The spleen serves as a major filter for circulating pathogens and is crucial in the clearance of systemically delivered oncolytic viruses, which potentially undermines the efficacy of virotherapy [20]. Given that OVM is sequestered in the spleen of various animal hosts [21], we sought to explore the role of the spleen in OVM treatment by performing splenectomy prior to orthotopic glioma cell implantation. Spleen removal had no effect on survival in untreated mice, whereas the antitumor activity of intravenous OVM was completely abrogated rather than potentiated after splenectomy (Fig. 2H). We then assessed T-cell kinetics in the periphery and TME. We found that the frequencies of total T cells, as well as those of the CD4^+^ and CD8^+^ T-cell subsets, were comparable in the blood and the TME between the asplenic and wild-type mice that did not receive OVM treatment (Fig. 2I, J). However, OVM failed to stimulate the expansion of T-cell populations in the peripheral blood of splenectomized mice (Fig. 2I). In the TME, OVM-induced increases in the frequencies of total T cells and CD4^+^ T-cell subpopulations were completely abolished in asplenic mice. Although an increase in tumor-infiltrating CD8^+^ T cells following OVM infusion occurred in splenectomized mice, this increase was much less prominent than that in their wild-type counterparts (Fig. 2J). Taken together, these results indicate that the spleen positively affects the OVM-induced T-cell response and is necessary for the therapeutic efficacy of OVM.

### Therapeutic efficacy of OVM against gliomas is mediated by splenic B cells

**Fig. 3:**
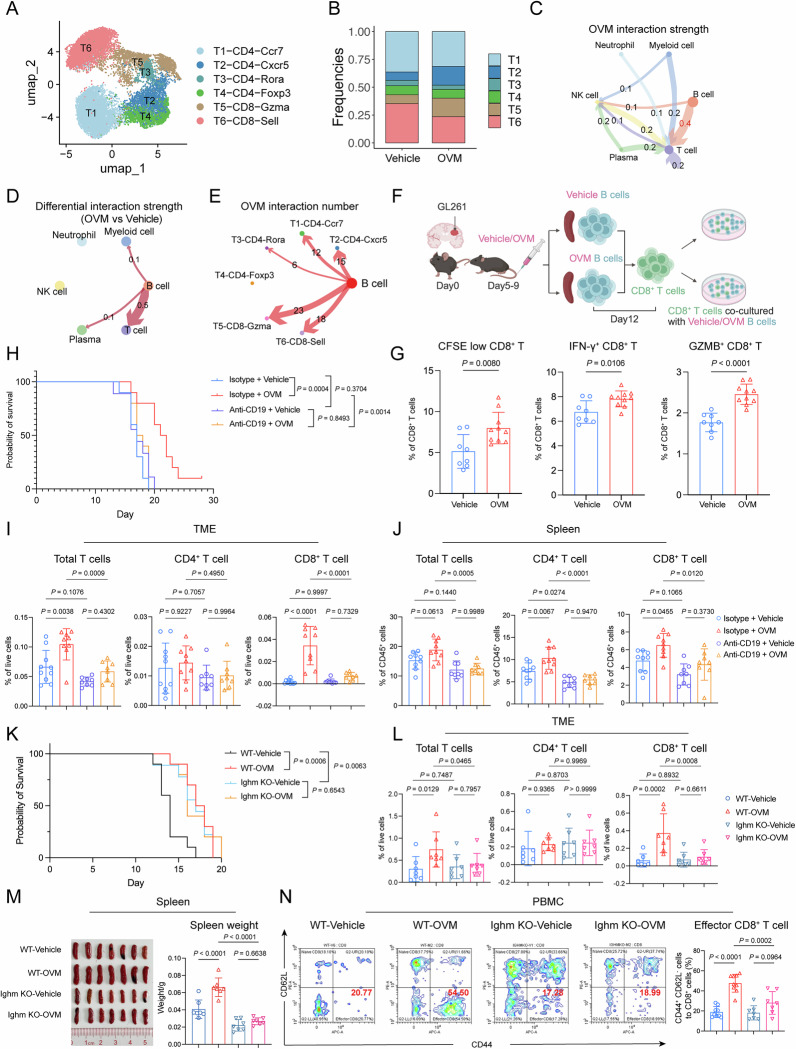
Splenic B cells are indispensable for the antiglioma activity of OVM. **A** Uniform manifold approximation and projection (UMAP) plot of splenic T-cell scRNA-seq data, showing six distinct subsets, with each dot representing a single cell colored by cluster. **B** Frequencies of the six T-cell subsets. **C** CellChat analysis illustrating the interaction strengths among major immune cell populations, including neutrophils, myeloid cells, NK cells, B cells, plasma cells, and T cells, in the OVM-treated group. **D** Differential interaction strengths between the OVM- and vehicle-treated groups across the indicated immune cell populations. **E** Interaction numbers between B cells and the six T-cell subsets in the OVM-treated group. **F** Schematic of the B-cell–CD8^+^ T-cell coculture experiment. GL261 cells were intracranially implanted on Day 0. The mice received daily intravenous injections of OVM or vehicle from Days 5–9. Splenic B cells were isolated on Day 12 and cocultured with tumor-derived CD8^+^ T cells at a 5:1 (B:T) ratio. **G** Proliferation of CD8^+^ T cells was assessed by CFSE dilution, and the proportions of IFN-γ^+^ and GZMB^+^ CD8^+^ T cells were quantified by flow cytometry. **H** Kaplan‒Meier survival curves of GL261 tumor-bearing mice receiving intraperitoneal injections of an anti-CD19 antibody or isotype control combined with intravenous injections of OVM or vehicle. **I**, **J** Quantification of total, CD4^+^, and CD8^+^ T cells in the TME and spleen of GL261 tumor-bearing mice across the four treatment groups. **K** Kaplan‒Meier survival curves of GL261 tumor-bearing C57BL/6J wild-type or Ighm-KO mice receiving vehicle or OVM treatment. Quantification of T-cell subsets in the TME (**L**), analysis of spleen morphology and weight (**M**), and the proportion of effector CD8^+^ T cells (CD44^+^ CD62L^−^) within total CD8^+^ T cells in the PBMCs (**N**) of GL261 tumor-bearing C57BL/6J WT or Ighm-KO mice

To elucidate the mechanisms underlying the spleen-dependent antiglioma effects of OVM, splenocytes were isolated from both OVM- and vehicle-treated glioma-bearing mice for single-cell RNA sequencing (scRNA-seq). After quality control, 43,926 cells were obtained for analysis. Six immune-related cell types were identified on the basis of canonical lineage markers via uniform manifold approximation and projection (UMAP) analysis combined with unsupervised clustering (Supplementary Fig. S3A). Among them, T cells and B cells (approximately 34% and 43%, respectively) outnumbered other immune subsets. Given that OVM induced an increase in tumor-infiltrating T lymphocytes, we focused on T-cell subsets. Six transcriptionally distinct T-cell clusters were identified on the basis of their gene expression profiles (Fig. 3A). Notably, the T2 cluster and T5 cluster were enriched in OVM-treated mice (Fig. 3B). The cells in the T2 cluster expressed transcripts encoding surface markers, cytokines and coactivators associated with T follicular helper (Tfh) cell function (Cxcr5, Bcl6, Pdcd1 and Icos). The T5 cluster displayed elevated expression of genes linked to CD8^+^ T-cell activation (Gzma, Gzmb and Gzmk). To determine the potential cellular subset that activates T cells, we performed intercellular communication analysis across 6 immune subpopulations via the CellChat pipeline. We found that all other 5 immune subpopulations interacted with T cells. The greatest strength of the predicted interaction was identified between B cells and T cells in the OVM-treated group (Fig. 3C). Furthermore, we explored the differences in the interaction patterns of splenic B cells between the OVM-treated and control groups. The strength of the predicted interaction between B cells and T cells was greater in OVM-treated mice than in control mice (Fig. 3D). We also investigated the interaction between B cells and individual T-cell clusters. The greatest number of predicted interactions between B cells and CD8^+^ T cells was observed following OVM treatment (Fig. 3E). To confirm the role of B cells in the activation of CD8^+^ T cells, B cells isolated from either the OVM-treated group or the vehicle-treated control group were cocultured with CD8^+^ T cells obtained from untreated glioma-bearing mice (Fig. 3F). We found that B cells from the OVM-treated group (B_OVM_) had a greater capacity to activate CD8^+^ T cells than did those from the vehicle-treated control group (B_veh_) (Fig. 3G). Moreover, increasing B-cell-to-T-cell ratios correlated with proportional increases in CD8^+^ T-cell subset frequencies (Supplementary Fig. S3B–D), indicating that B-cell-mediated activation was dose dependent. Given that DCs are specialized antigen-presenting cells (APCs) that link innate and adaptive immunity [22], we also explored the impact of OVM on the antigen-presenting function of DCs. In contrast to B cells, DCs from OVM-treated mice did not exhibit an increased capacity to promote CD8^+^ T-cell proliferation and activation compared with those from control mice (Supplementary Fig. S3E). Taken together, these results suggest that OVM activated CD8^+^ T cells via B–T-cell interactions.

To determine the requirement of B cells, we first injected mice intraperitoneally with an anti-CD19 antibody before and after intracranial glioma implantation to deplete B cells (Supplementary Fig. S3F). As shown in Supplementary Fig. S3G, H, administration of the anti-CD19 antibody efficiently removed B cells from the spleen and peripheral blood but spared B cells in the TME. OVM failed to prolong the survival of glioma-bearing mice after B-cell depletion (Fig. 3H). The expansion of T-cell populations in the blood and increased infiltration of T lymphocytes in the TME did not occur following OVM administration (Fig. 3I, J and Supplementary Fig. S3I). For further validation, Ighm-knockout (Ighm-KO) mice, which are deficient in mature B cells, were used. Intriguingly, compared with wild-type control Ighm-KO mice, glioma-bearing Ighm-KO mice presented a survival advantage but failed to respond to OVM treatment (Fig. 3K). OVM-induced reversal of immunosuppression, including spleen atrophy, lymphopenia and T-cell exclusion, was not observed in Ighm-KO mice (Fig. 3L–N and Supplementary Fig. S3J, K). Collectively, these data indicate that splenic B cells intensively interact with T cells and play crucial roles in the proliferation and activation of CD8^+^ T cells after OVM treatment.

### Direct interaction of OVM-induced B–T cells requires immune synapses

**Fig. 4:**
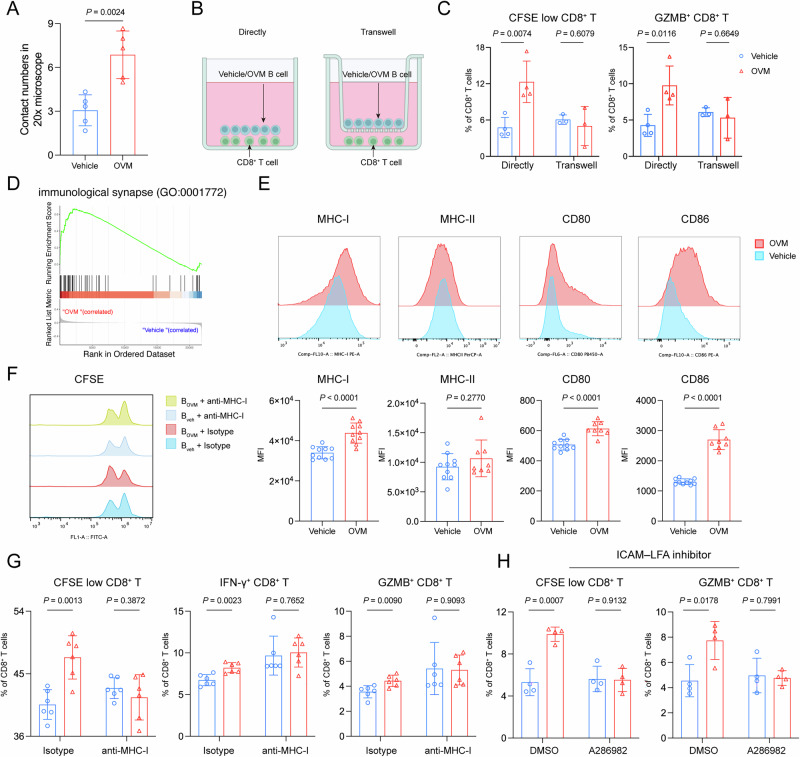
Immune synapses mediate direct B–T-cell interactions after OVM treatment. **A** Quantification of direct contacts between B cells and CD8^+^ T cells via live-cell imaging. The number of B–CD8^+^ T-cell contacts was counted in 20× microscope fields. **B** Splenic B cells were isolated from GL261 tumor-bearing mice treated with vehicle or OVM and cocultured with tumor-derived CD8^+^ T cells at a 5:1 (B:T) ratio either in direct contact or separated by a transwell insert. **C** CD8^+^ T-cell proliferation was measured by CFSE dilution, and the frequency of GZMB^+^ CD8^+^ T cells was determined by flow cytometry. **D** Gene set enrichment analysis (GSEA) showing enrichment of the immunological synapse pathway (GO:0001772) in B cells following OVM treatment. **E** Representative histograms and corresponding quantification of MHC-I, MHC-II, CD80, and CD86 expression on splenic B cells. **F** Representative CFSE histograms of CD8^+^ T cells cocultured with B_OVM_ or B_veh_ cells in the presence of an anti-MHC-I antibody or an isotype control (100 µg/mL). **G** Quantification of CD8^+^ T-cell proliferation by CFSE dilution and the proportions of IFN-γ^+^ and GZMB^+^ CD8⁺ T cells. **H** CD8^+^ T-cell proliferation and activation in B-cell coculture in the presence of 1 µM ICAM1–LFA inhibitor A286982. The proportions of CFSE-low CD8^+^ T cells and GZMB^+^ CD8^+^ T cells among total CD8^+^ T cells were measured via flow cytometry

For real-time visualization of B-cell–CD8^+^ T-cell interactions, the two cell populations were labeled with Cy5 (B cells) and CFSE (CD8^+^ T cells) and subjected to live-cell imaging. Imaging analysis revealed that, compared with B_veh_ cells, B_OVM_ cells had more frequent physical contact with CD8^+^ T cells (Fig. 4A and Supplementary Fig. S4A). To assess whether these interactions require direct cell–cell contact, transwell chambers were used to physically separate CD8^+^ T cells and B cells. As shown in Fig. 4B, C, proliferation and activation were observed in CD8^+^ T cells that were in direct contact with B_OVM_ cells but not in those in the transwell-separated group. Biological function annotation analysis revealed that genes associated with immune synapses were enriched in B cells after OVM treatment (Fig. 4D). The immune synapse is a specialized cell junction that integrates TCR–peptide–MHC cognate binding, adhesion molecule pairs and costimulatory signals together for antigen presentation [23]. Flow cytometry analysis revealed that B_OVM_ cells had greater expression of MHC-I, CD80 and CD86 on their surface than did B_veh_ cells, whereas no difference in the level of MHC-II was observed (Fig. 4E). The presence of an MHC-I blocking antibody in the coculture abrogated B_OVM_ cell-induced proliferation and activation of CD8^+^ T cells (Fig. 4F, G). In addition, productive engagement of intercellular adhesion molecule 1 (ICAM1) on APCs with lymphocyte function-associated antigen (LFA) on T lymphocytes results in peripheral supramolecular activation clusters (pSMACs) within immune synapses [24]. We found that ICAM1–LFA inhibition efficiently disabled the interaction between B_OVM_ and CD8^+^ T cells (Fig. 4H). Together, these results indicate that OVM induces direct contact between B cells and T cells through immune synapses.

### B_OVM_ cells activate CD8^+^ T cells via antigen cross-presentation

**Fig. 5:**
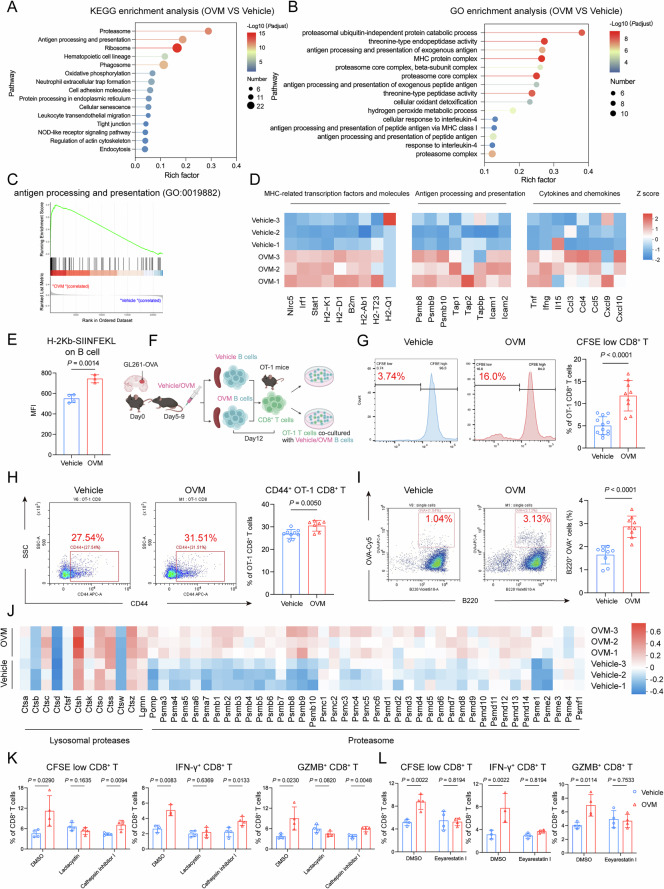
B cells activate CD8^+^ T cells via antigen cross-presentation following OVM administration. Bubble plots of KEGG (**A**) and GO (**B**) pathway enrichment for DEGs in B cells following OVM versus vehicle treatment. **C** GSEA of B cells showing upregulation of the antigen processing and presentation pathway (GO:0019882) in B_OVM_ cells compared with B_veh_ cells. **D** Heatmap showing the average expression of MHC-related transcription factors and molecules, antigen processing and presentation, and cytokines and chemokines in B_veh_ and B_OVM_ cells. **E** Flow cytometric analysis of surface H-2Kb-SIINFEKL expression on B cells, with the results shown as the MFI. **F**–**H** B_veh_ and B_OVM_ cells were isolated from the spleens of GL261-OVA tumor-bearing mice treated with vehicle or OVM and cocultured with OT-1 CD8^+^ T cells (**F**). OT-1 CD8^+^ T-cell proliferation was measured by CFSE dilution (**G**), and activation was assessed by the expression of CD44 (**H**). **I** B_veh_ and B_OVM_ cells were incubated with OVA-Cy5 (10 µg/mL) for 24 h, and the proportion of OVA^+^ B cells was determined. **J** Heatmap showing the average expression of lysosomal protease- and proteasome-associated genes in B_veh_ and B_OVM_ cells. B_veh_ and B_OVM_ cells were pretreated with the proteasome inhibitor lactacystin (2.5 µg/mL), the lysosome inhibitor cathepsin inhibitor I (10 µM) (**K**), or the ERAD inhibitor eeyarestatin I (3 µM) (**L**) before being cocultured with CD8^+^ T cells from tumor-bearing mice. CD8^+^ T-cell proliferation and the frequencies of IFN-γ^+^ and GZMB^+^ CD8^+^ T cells were then assessed via flow cytometry

Given the critical role of immune synapses in the interaction between B_OVM_ cells and CD8^+^ T cells, we asked whether antigen cross-presentation accounts for the B-cell-induced activation of CD8^+^ T cells. To this end, we investigated pathways enriched in B cells by analyzing the differentially expressed genes (DEGs) from our scRNA-seq dataset. KEGG and GO analyses revealed that pathways related to antigen take-up, processing and presentation were enriched in B_OVM_ cells (Fig. 5A–C). Specifically, we found that genes associated with MHC components and transcription factors, antigen processing and adhesion molecules, and cytokines and chemokines were upregulated in B_OVM_ cells (Fig. 5D). These results suggest that B cells cross-present antigens to CD8^+^ T cells following OVM treatment. To determine the relevance and specificity of antigen cross-presentation, we isolated splenic B cells from GL261-OVA tumor-bearing mice treated with either OVM or vehicle. Flow cytometry analysis revealed that B_OVM_ cells had elevated expression of the MHC-I-restricted OVA epitope H-2Kb-SIINFEKL on their surface (Fig. 5E). When cocultured with OVA-specific OT-1 CD8^+^ T cells, B_OVM_ cells were more efficient at promoting the proliferation and activation of OT-1 cells (Fig. 5F–H). Given that antigen uptake is the initial step in antigen-presenting cascades, we evaluated the capacity of B cells to acquire exogenous peptide antigens by pulsing them with Cy5-labeled OVA. A significant increase in the frequency of B cells positive for Cy5 was found in the OVM-treated group (Fig. 5I), indicating enhanced antigen capture. Proteasomes and lysosomes are two major compartments for the processing of peptide antigens. We found that B_OVM_ cells presented markedly elevated expression of genes associated with proteasomes rather than lysosomes (Fig. 5J). Blockade of proteasome activity but not lysosomal function completely abolished the subsequent proliferation and activation of CD8^+^ T cells (Fig. 5K). This finding indicated that B_OVM_ cells rely on the proteasome-dependent cytosolic pathway to process peptide antigens, which are then transported to the endoplasmic reticulum (ER) for assembly with MHC I molecules. When exposed to an ER-associated degradation (ERAD) inhibitor, B_OVM_ cells lost their ability to activate CD8^+^ T cells (Fig. 5L). Taken together, these results indicate that antigen cross-presentation via the cytosolic pathway is responsible for the B-cell-elicited activation of CD8^+^ T cells following OVM treatment.

### An enriched Bst2^+^ B-cell subset with superior cross-priming capacity contributes to OVM-elicited adaptive immunity against gliomas

**Fig. 6:**
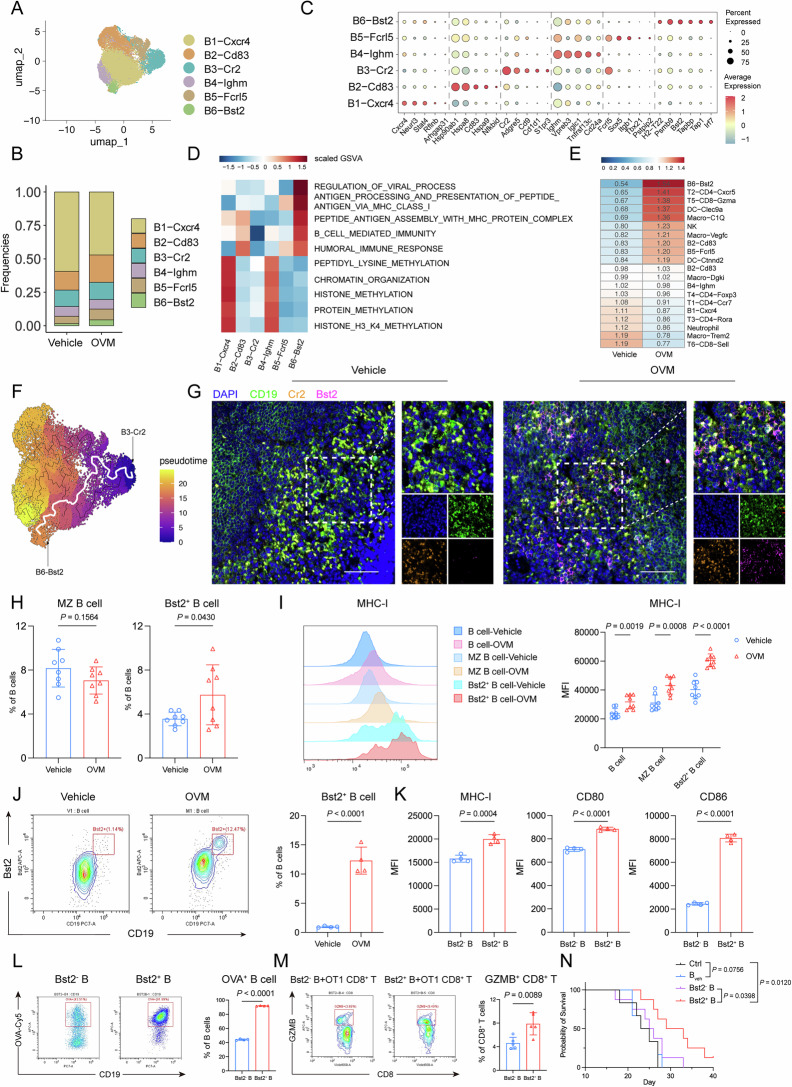
Bst2^+^ B cells are enriched and exhibit enhanced antigen cross-presentation. **A** UMAP plot of B cells showing further subdivision into 6 distinct subsets. **B** Quantification of the frequencies of B-cell subsets. **C** Representative marker genes for each subset are shown in a bubble plot, with bubble size representing the percentage of cells expressing each gene and color indicating the average expression level. **D** Heatmap showing the GSVA results across B-cell subsets. **E** Enrichment analysis of immune cell subsets on the basis of *Ro/e*, showing the distribution preference of each cluster. The *Ro/e* ratio was calculated as the observed number of cells divided by the expected number in the OVM-treated and control groups. **F** Pseudotime analysis of B-cell subsets, with color indicating the differentiation stage. **G** Representative images of immunofluorescence staining for Bst2^+^ B cells in the spleen. Scale bar, 50 µm. **H** Flow cytometric analysis of splenic MZ B cells and Bst2^+^ B cells in glioma-bearing mice. **I** Representative histograms and statistical summary of surface MHC-I expression on Bst2^+^ B cells and MZ B cells. Sorted splenic MZ B cells from glioma-bearing mice were treated with OVM, the proportion of Bst2^+^ B cells was measured (**J**), and the surface expression of MHC-I, CD80, and CD86 on these cells was assessed (**K**). **L** The OVA uptake capacity of Bst2^+^ B cells was evaluated via flow cytometry. **M** OT-1 CD8^+^ T cells were cocultured with Bst2^+^ or Bst2^−^ B cells, and the proportion of GZMB^+^ OT-1 CD8^+^ T cells was assessed via flow cytometry. **N** B_veh_ cells, Bst2^−^ B cells and Bst2^+^ B cells were adoptively transferred into B-cell-deficient mice bearing orthotopic GL261-OVA gliomas. Ctrl: Mice receiving PBS served as the control group. B_veh_: B cells sorted from GL261-OVA mice treated with vehicle

To identify the specific B-cell subset responsible for mediating antigen cross-presentation and subsequent CD8^+^ T-cell activation following OVM treatment, we extracted 19,031 B cells from our scRNA-seq dataset and performed UMAP analysis combined with unsupervised clustering. Six transcriptionally distinct subclusters were defined (Fig. 6A). Among them, the frequencies of the B2 cluster, B5 cluster, and B6 cluster increased significantly in the OVM-treated group (Fig. 6B). The B2 cluster presented upregulated expression of genes associated with activated B cells (Hsp family genes and Cd83). The B5 cluster was defined by the expression of Fcrl5, a marker for memory B cells. The B6 cluster was positive for the surface marker Bst2 and highly expressed H2-T22, Tap1 and Irf7 (Fig. 6C). Functional pathway analysis revealed that pathways associated with MHC class I loading complex assembly and antigen cross-presentation were predominantly enriched in the Bst2^+^ B-cell subset (Fig. 6D). To determine the distribution preference of immune cell subpopulations, we quantified the enrichment of individual immune cell subsets on the basis of the *Ro*/*e* analysis, by which the ratio of observed to expected cell numbers for each cell cluster in the OVM-treated group and controls was calculated. We found that Bst2^+^ B cells were among the top-ranked immune cells that were preferentially enriched in the OVM-treated group (Fig. 6E). Next, we sought to determine the origin of Bst2^+^ B cells. Dynamic trajectory inference of B-cell ontogeny via pseudotime analysis strongly suggested that Bst2^+^ B cells originated from the B3 cluster (Fig. 6F and Supplementary Fig. S5A–E), which corresponds to marginal zone (MZ) B cells characterized by increased expression of Cr2, CD9 and CD1d. In the spleens of glioma-bearing mice, Bst2^+^ B cells were enriched and codistributed with MZ B cells after OVM treatment (Fig. 6G). Notably, OVM treatment specifically increased the proportion of splenic Bst2^+^ B cells rather than MZ B cells (Fig. 6H). We found that genes associated with antigen processing and presentation pathways were enriched in Bst2^+^ B cells compared with MZ B cells (Supplementary Fig. S6A–C). Flow cytometry analysis revealed that Bst2^+^ B cells had a greater baseline level of MHC-I molecules and more significant upregulation of MHC-I molecules following OVM treatment than did the total B-cell population and the MZ B-cell subset (Fig. 6I). Given the suggested origin of Bst2^+^ B cells from MZ B cells and the critical role of MZ B cells in the first-line response to blood-borne pathogens in the spleen, we wondered whether Bst2^+^ B cells can be expanded from MZ B cells ex vivo. MZ B cells were thus isolated from glioma-bearing mice and stimulated with OVM for 48 h. In OVM-treated MZ B cells, the percentage of Bst2^+^ cells markedly increased from 1% to 12% (Fig. 6J). Flow cytometry analysis revealed significantly elevated levels of MHC-I molecules and costimulatory factors (CD80 and CD86) in Bst2^+^ B cells (Fig. 6K). When cocultured with autologous CD8^+^ T cells, Bst2^+^ B cells prominently enhanced the proliferation of IFN-γ^+^ CD8^+^ T cells (Supplementary Fig. S7A–C). In the GL261-OVA murine model, ex vivo-expanded Bst2^+^ B cells demonstrated a superior capacity for OVA antigen uptake (Fig. 6L) and induced robust activation of antigen-specific OT-1 CD8^+^ T cells compared with Bst2^−^ B cells (Fig. 6M). To determine the therapeutic relevance of Bst2^+^ B cells, we conducted adoptive transfer experiments in a B-cell-deficient mouse model. Splenic MZ B cells isolated from wild-type GL261-OVA-bearing mice were stimulated with OVM in vitro and sorted according to their Bst2 expression status. Bst2^+^ and Bst2^−^ B cells were transferred to GL261-OVA-bearing Ighm-KO mice. Notably, only the transfer of Bst2^+^ B cells significantly prolonged the survival of B-cell-deficient mice, whereas neither Bst2^−^ B cells nor B cells from vehicle-treated GL261-OVA-bearing controls improved survival (Fig. 6N). These findings indicate that OVM-induced Bst2^+^ B cells are crucial for tumor antigen cross-presentation and are efficient in eliciting adaptive immunity against GBMs.

### OVM treatment sensitizes intracranial gliomas to anti-PD-1 therapy

**Fig. 7:**
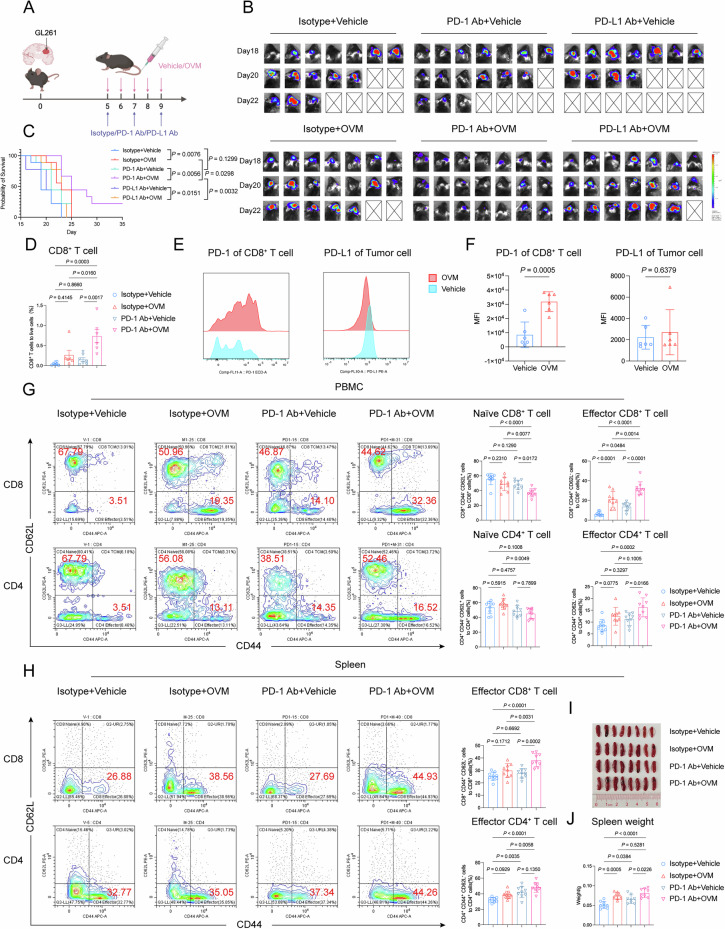
OVM treatment sensitizes intracranial gliomas to anti-PD-1 therapy. **A** Schematic of the combination therapy treatment schedule. **B** Representative bioluminescence images of GL261-Luc tumors acquired via the IVIS Spectrum system at the indicated time points. **C** Overall survival of GL261-Luc orthotopic tumor-bearing mice in the isotype + vehicle, isotype + OVM, PD-1 Ab + vehicle, PD-1 Ab + OVM, PD-L1 Ab + vehicle and PD-L1 Ab + OVM groups was assessed via Kaplan‒Meier survival curves. **D** Flow cytometric quantification of CD8^+^ T-cell numbers within the TME of GL261 tumor-bearing mice. **E** Representative flow cytometric histograms showing the MFI of PD-1 on CD8^+^ T cells and PD-L1 on tumor cells. **F** Quantitative analysis of the PD-1 MFI on CD8^+^ T cells and the PD-L1 MFI on tumor cells in the TME. **G** Flow cytometric analysis of the proportions of naïve CD8^+^ T cells, effector CD8^+^ T cells, naïve CD4^+^ T cells, and effector CD4^+^ T cells among the PBMCs of GL261 tumor-bearing mice. **H** Flow cytometric quantification of the proportions of effector CD4^+^ T cells and effector CD8^+^ T cells in the spleens of GL261 tumor-bearing mice. **I** Representative gross images of spleens from GL261 tumor-bearing mice. **J** Quantitative analysis of the spleen weights

To date, ICB fails to provide clinical benefits in most patients with GBM. Various strategies to reverse the resistance of GBMs to ICB have been extensively investigated [25–27]. In the present study, we explored whether OVM could sensitize gliomas to PD-1/PD-L1 blockade (Fig. 7A). Our results demonstrated that GL261 cells were refractory to anti-PD-1 and anti-PD-L1 therapy in vivo (Fig. 7B, C). Intravenous OVM delivery sensitized intracranial gliomas to PD-1 inhibitors but not to PD-L1 blockade (Fig. 7B, C). Compared with monotherapy, the combination of OVM and a PD-1 inhibitor significantly promoted the recruitment of CD8^+^ T cells into the TME and markedly suppressed intracranial glioma growth (Fig. 7D). We profiled the expression of PD-1/PD-L1 in the TME and found that OVM treatment significantly increased the mean fluorescence intensity (MFI) of PD-1 on CD8^+^ T cells, whereas no significant change in PD-L1 expression on tumor cells was detected (Fig. 7E, F). In addition, the combination of OVM and a PD-1 inhibitor significantly increased the frequencies of effector CD4^+^ and CD8^+^ T cells in peripheral blood, along with a concomitant and significant reduction in naïve T lymphocytes (Fig. 7G), which suggested the differentiation and activation of peripheral T cells. Moreover, OVM combined with a PD-1 inhibitor facilitated the expansion of splenic effector CD4^+^ and CD8^+^ T cells (Fig. 7H), accompanied by the restoration of splenic size and weight (Fig. 7I, J). Collectively, these findings indicate that OVM synergizes with PD-1 inhibitors for glioma treatment by reshaping systemic immunity and the local immune environment.

## Discussion

Glioblastoma multiforme (GBM) is the most aggressive primary brain tumor with a grim prognosis, and standard therapies and ICB fail to achieve curative effects. The major hurdle undermining ICB efficacy in GBM stems from the profoundly immunosuppressive microenvironment [28]. Oncolytic virotherapy has emerged as a promising immunotherapeutic strategy for GBM. As highlighted by a comprehensive review [29], oncolytic viruses (OVs) reshape the GBM microenvironment mainly through the following three mechanisms. First, OVs replicate selectively within neoplastic cells, leading to immunogenic cell death that activates innate immunity through pathogen-associated molecular pattern (PAMP) receptors. Second, oncolysis-facilitated release of tumor-associated antigens (TAAs) recruits APCs to the TME and induces adaptive antitumor responses. Moreover, OVs induce the secretion of proinflammatory cytokines, which renders gliomas more recognizable to the immune system by enhancing MHC expression on both infected and bystander tumor cells. In phase I–II clinical trials, locoregionally delivered OVs demonstrated favorable tolerability and ability to modulate the tumor microenvironment and trigger antitumor immune responses against GBMs. Notably, subsets of patients receiving OV-based therapy achieve long-term survival.

**Fig. 8:**
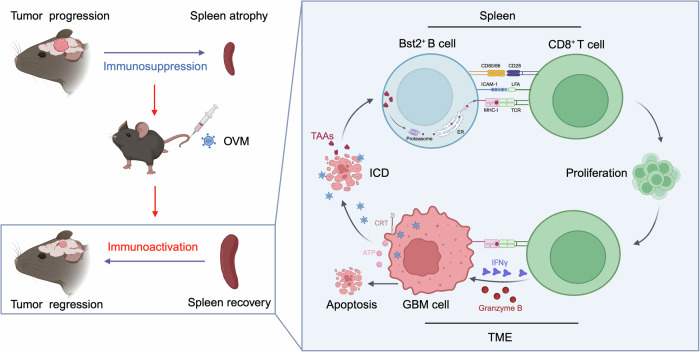
Schematic diagram showing the proposed mechanism responsible for OVM-induced adaptive immunity against GBMs. GBM induces splenic atrophy and peripheral immunosuppression, whereas OVM treatment reverses splenic atrophy and restores peripheral immune function. Systemic administration of OVM directly lyses GBM cells and triggers immunogenic cell death. OVM promotes the expansion of the splenic B-cell subset positive for Bst2, which processes tumor-derived antigens through the cytosolic pathway. Bst2^+^ B cells upregulate MHC-I and costimulatory factors and prime tumor-specific CD8^+^ T cells via immune synapses. Activated CD8^+^ T cells infiltrate and eliminate glioma cells in the TME

Currently, extensive research efforts are focused on OVs leveraging various viral vector platforms and delivery strategies for GBM therapy. In this study, we found that a novel oncolytic virus, OVM, which can be repeatedly intravenously injected, efficiently repressed orthotopic glioma growth and synergized with anti-PD-1 therapy. OVM activated antitumor immunity through inducing the immunogenic death of glioma cells and promoting antigen cross-presentation by a subset of Bst2^+^ B cells in the spleen (Fig. 8). Our findings indicate the feasibility of intravenous OVM for GBM treatment and unveil a novel oncolytic virus-mediated antitumor immunostimulatory mechanism, which provides an alternative immunotherapeutic strategy to overcome GBM-induced immunosuppression.

Impaired antigen presentation constitutes a primary impediment to efficacious immunotherapy for malignant gliomas. The immunosuppressive milieu of GBMs severely impairs antigen presentation through multiple mechanisms. Glioma-derived factors (e.g., TGF-β, IL-10, and PGE2) suppress DC activation and inhibit the production of crucial immunostimulatory cytokines such as IL-12 [30]. Moreover, glioma-associated extracellular matrix components (e.g., hyaluronan) and metabolites (e.g., lactic acid, kynurenine) mediate DC apoptosis [31, 32]. In addition to deficits in DC function, tumor-associated macrophages (TAMs) manipulate antigen presentation to drive cognate tumor-specific CD8^+^ T cells toward terminal exhaustion [33]. Importantly, our study revealed that OVM-induced extratumoral antigen cross-presentation is mediated by splenic B cells. Intravenous OVM delivery redirected tumor antigen presentation to the spleen and induced splenic B cells into potent APCs that engage in antigen presentation via MHC-I complexes to CD8^+^ T cells. This alternative B-cell-dependent extratumoral antigen processing and T-cell priming overcomes the deficits in intratumoral antigen presentation.

The spleen is the largest secondary lymphoid organ that actively shapes systemic antitumor immunity in a paradoxical context-dependent manner. The spleen undergoes cellular and metabolic reconfiguration during tumor progression. Splenomegaly with extramedullary hematopoiesis triggered by tumor cells expands myeloid suppressors via GM-CSF/CCL2-dependent pathways, facilitating immunosuppression and tumor progression in hepatocellular carcinoma [34]. In breast cancer, splenic stromal cells recruit glycolytic neutrophils that establish a glucose-deprived environment and induce T-cell anergy by impairing STAT5 signaling [35]. Conversely, the spleen can also play a crucial role in coordinating robust antitumor immunity in immunotherapy. A recent finding by Morgan et al. revealed an anatomically related differentiation trajectory of exhausted CD8^+^ T cells. Intermediate-exhausted T cells that dictate the response to ICB are enriched in the white pulp of the spleen. In contrast, tumors harbor increased frequencies of terminally exhausted T cells, and draining lymph nodes exhibit early-stage exhausted phenotypes [36]. In the present study, our data unequivocally indicate that the spleen is indispensable for OVM-induced antiglioma immunity. Splenectomy completely abolished the therapeutic effects of OVM and the infiltration of T cells into the TME. Single-cell RNA sequencing revealed remodeling of the splenic immune landscape after OVM treatment, highlighting the enhanced interaction between B cells and T cells. The spleen provides a sanctuary site where B cells cross-present tumor antigens, which spatially decouples T-cell priming from the immunologically hostile TME. The spleen is therefore a remote hub that orchestrates OVM-induced antiglioma immunity and is a prerequisite for the therapeutic efficacy of OVM.

The antitumor role of B cells is controversial. The abundance of B cells in the tumor and blood of head and neck squamous-cell carcinoma (HNSCC) patients has been linked to an immunologically hot tumor microenvironment and represents a master predictor of the ICB response [37]. Similarly, the presence of B cells clustered with T cells in tertiary lymphoid structures (TLSs) is associated with enhanced immunotherapy efficacy and favorable clinical outcomes in various malignancies [38]. The production of antitumor antibodies and the activation of CD4^+^ T cells through antigen presentation account for the immunomodulatory ability of B cells in TLSs [39, 40]. However, infiltrating B cells in GBMs are inclined to adopt an immunosuppressive phenotype in response to environmental cues. Naïve B cells recruited to the TME are educated and converted into regulatory B cells (Bregs) by microvesicles and cytokines derived from glioma cells and myeloid-derived suppressor cells (MDSCs) [41, 42]. GBM-associated Bregs upregulate the inhibitory molecules CD155 and PD-L1 to suppress T-cell activity. Intratumoral depletion of B cells via local delivery of an anti-CD20 antibody extends survival in orthotopic GBM mouse models [43]. In line with that finding, we showed that systemic B-cell deficiency in GBM-bearing Ighm-KO mice conferred a survival advantage over wild-type controls, whereas peripheral administration of an anti-CD19 antibody, which spared some intratumoral B cells, failed to provide the same therapeutic benefit. In addition to Bregs, intratumoral plasma cells promote GBM progression. In a recent study, the authors demonstrated that plasma cells migrate to the glioma stem cell (GSC) niche, promoting GSC proliferation via IgG secretion and activation of the FcgRIIA-AKT-mTOR pathway [44]. The ability of B cells to modulate the antitumoral immune response makes them promising candidates for the development of immunotherapies for GBM. Reversing the suppressive phenotype of Bregs through the blockade of inhibitory signals or the use of B-cell-based neoantigen vaccines that activate autologous CD8 + T cells has been shown to increase ICB efficacy [42, 45]. In the present study, we found that OVM reinstated the antigen-presentation machinery of B cells. A subset of splenic Bst2^+^ B cells was enriched and upregulated MHC-I and costimulatory molecules, which activated cognate CD8^+^ T cells and repressed glioma progression. Moreover, these Bst2^+^ B cells with superior cross-priming capacity can be readily expanded ex vivo upon OVM stimulation and improve the survival of GBM-bearing mice. Our findings support the translational potential of Bst2^+^ B cells as a novel platform for personalized B-cell-based neoantigen vaccines for GBM patients.

GBMs are universally unresponsive to ICB-based immunotherapy, primarily due to the exclusion and functional exhaustion of T lymphocytes within the TME. Systemic immunosuppression driven by GBM further contributes to treatment resistance. Specifically, the impairment of the total lymphocyte pool in GBM patients exacerbates the scarcity of TILs. Consistent with clinical observations in GBM patients, intracranial tumor-bearing mice recapitulate hallmark features of systemic immunosuppression, including splenic atrophy and peripheral lymphopenia, and fail to respond to monotherapy with PD-1 or PD-L1 inhibitors. Notably, the combination of OVM and PD-1 inhibitors overcomes therapeutic resistance through complementary mechanisms. First, OVM reversed GBM-induced peripheral T-cell depletion and enhanced the infiltration of lymphocytes into the TME. Second, OVM treatment upregulated PD-1 expression on CD8^+^ T cells within the TME, which is likely a compensatory negative regulatory mechanism triggered by increased CD8^+^ T-cell activation and sustained exposure to tumor antigens in the TME. The use of a PD-1 inhibitor abrogated this inhibitory signaling axis and mitigated CD8^+^ T-cell exhaustion, thereby maximizing the antitumor effects. Collectively, our findings position OVM as a promising immune sensitizer that potentiates the therapeutic efficacy of PD-1 inhibitors against GBM.

While OVs are well documented to activate broad antitumor immunity [46, 47], concerns regarding their potential to induce allergic reactions remain relevant for clinical translation. A systematic analysis of the literature revealed that allergic reactions associated with OVs are rare and typically mild, manifesting as local injection-site erythema or transient pruritus [48]. No severe anaphylactic reactions have been reported in glioma-related OV trials (e.g., DNX-2401, PVSRIPO, and HSV-G47Δ) [5, 6, 49]. A recent clinical study of OVM in HCC patients (NCT04665362) demonstrated the favorable safety profile of OVM for allergies [10]. A comprehensive safety analysis of 13 patients with HCC revealed that the most frequent adverse events (AEs) were influenza-like symptoms and transient cytopenia. Allergy-related AEs were absent throughout the treatment course. Critically, an ongoing phase I/II clinical trial (NCT07093814), which aims to evaluate the safety and efficacy of OVM for recurrent or progressive GBMs, will provide direct evidence to clarify the potential association between OVM and allergic reactions in glioma patients.

Our study has several limitations. First, the reliance on murine glioma models may not fully recapitulate the complex immune microenvironment and intertumoral heterogeneity of human GBMs. The efficacy of OVM across distinct GBM subtypes remains to be defined. Second, the molecular mechanism underlying the OVM-induced enrichment of splenic Bst2^+^ B cells is unresolved and requires further investigation. Additionally, preclinical data and early-phase trials in HCC patients have demonstrated the safety of intravenous OVM administration, but uncertainties over potential off-target risks specific to GBM patients persist. OVM-related toxicity must be rigorously monitored and addressed in clinical trials for GBM.

In conclusion, our study proposes a novel oncolytic virus, OVM, as a potent activator of host antitumor immunity and an enhancer of ICB efficacy against GBMs. We revealed a previously unrecognized mechanism by which OVM reverses GBM-induced systemic immunosuppression and promotes CD8^+^ T lymphocyte infiltration within the TME through enhanced antigen cross-presentation by splenic Bst2^+^ B cells. The intravenous delivery route of OVM and its ability to cross the BBB make it readily applicable for clinical translation in GBM treatment.

## Materials and methods

### Cell lines

GL261 was a kind gift from Professor Chunsheng Kang at Tianjin Medical University. CT2A was a kind gift from Professor Lei Zhang at Shaanxi Normal University. U-118 MG, U-87 MG, Vero and BHK-21 cells were purchased from ATCC. GL261-Luc and GL261-OVA cells were generated from GL261 cells via transduction of Luc-loaded lentivirus (Obio Technology Co. Ltd., Shanghai, China) and OVA-loaded lentivirus (Obio Technology Co. Ltd., Shanghai, China), respectively. The cells were grown in Eagle’s minimum essential medium (Corning, 10-010-CV) or Dulbecco’s modified Eagle’s medium (Gibco, 11965092) supplemented with 10% fetal bovine serum (FBS; 10099-141, Gibco, USA) and 1% penicillin/streptomycin (SV30010, HyClone). All the cells were cultured at 37 °C in 5% CO_2_.

### Animals

The mice used in this study were 6- to 8-week-old females. C57BL/6J mice were purchased from Guangdong Medical Laboratory Animal Center. BALB/c-nu/nu mice were purchased from Guang Dong GemPharmatech Co., Ltd. C57BL/6JGpt-*H11*^em1Cin (Tcra&Tcrb)^/Gpt (OT-1) and B6/JGpt-Ighmem1Cd/Gpt (Ighm-KO) mice were purchased from GemPharmatech Co., Ltd. All the mice were bred in specific pathogen-free facilities. All the animal studies were approved by the Animal Ethical and Welfare Committee of Sun Yat-sen University.

### OVM production and quantification

OVM-c6v1, OVM-GFP and OVM-iRFP were propagated in Vero cells and provided by Guangzhou Virotech Pharmaceutical Technology. The virus titer was determined via a CCID_50_ assay in BHK-21 cells.

### Cell viability assay

Glioma cells were seeded into 48-well plates at a density of 10^4^ cells per well. Following overnight adherence, the cells were treated with OVM at MOIs of 0.001, 0.01, 0.1, 1, and 10. The samples were harvested at 24, 48, 72, and 96 h postinfection for analysis. The medium was aspirated and replaced with 200 µL of 3-(4,5-dimethylthiazol-2-yl)2,5-diphenyltetrazolium bromide (MTT; MP Biomedicals, 102227), 0.5 mg/mL final concentration, per well. After 2–4 h of incubation at 37 °C, the supernatant was carefully removed, and the MTT formazan precipitate was dissolved in 200 µL of dimethyl sulfoxide (DMSO; Sigma-Aldrich, 67-68-5). The absorbance was measured at 490 nm via a microplate reader (Synergy H1, BioTek).

### ATP assay

Cell culture supernatants were obtained and centrifuged at 1000 × *g* for 10 min at 4 °C to remove cell debris. The concentrations of ATP were measured with ELISA kits (Beyotime Biotechnology, S0027) in accordance with the manufacturer’s instructions.

### Tumor processing and ex vivo functional testing

This study was approved by the Institutional Review Board of Sun Yat-Sen University Cancer Center. GBM samples were freshly acquired during surgical resection. Live tumor tissues were minced into appropriately sized microtissue fragments (approximately 1 mm³) and uniformly seeded into 24-well plates. The plates were then incubated in a CO_2_ incubator. After 24 h of culture, the samples were treated with OVM (150 µL, titer: 1.16 × 10^8^ CCID_50_/mL), TMZ (50 mg/mL) or vehicle control for 72 h. Subsequently, MTT stock solution was added to each well at a volume equivalent to 10% of the total well volume. After 2–4 h of incubation at 37 °C, the tissue fragments were analyzed via a tissue cell image analyzer. The analyzer captured both transmitted light images of the tissue fragments and quantified the area stained with formazan blue. The rate of drug inhibition was calculated via the following formula:

$${{{\rm{Inbibition}}}}\left( \% \right)=\left(1-\frac{{{{\rm{BA}}}}-{{{\rm{treated}}}}{{{\rm{sample}}}}/{{{\rm{A}}}}-{{{\rm{treatedsample}}}}}{{{{\rm{BA}}}}-{{{\rm{control}}}}{{{\rm{sample}}}}/{{{\rm{A}}}}-{{{\rm{controlsample}}}}}\right)\times 100 \%$$

(A: Total area of tissue fragments in transmitted light images prior to drug treatment. BA, blue area, area stained with formazan blue in transmitted light images after drug treatment).

### qPCR

For the OVM replication assay, total RNA was extracted from tissue samples via TRIzol™ reagent (Thermo Fisher Scientific, 15596018CN). Viral RNA was quantified via one-step reverse transcription quantitative PCR (RT‒qPCR) via the FastKing One Step RT‒qPCR Kit (Probe, Tiangen Biotech, FP314-01) on an Applied Biosystems 7500 Fast Real-Time PCR System. A TaqMan® primer-probe set targeting the gene encoding nonstructural protein 2 (NSP2) of the OVM virus (designed and synthesized by Guangzhou Virotech Pharmaceutical Technology, Guangdong, China) was employed. To account for the nonspecific adsorption of viral particles to tissues, equal amounts of inactivated OVM-c6v1 were added to parallel samples as background controls. Viral replication within each tissue type was expressed as a replication coefficient, which was calculated as the ratio of viral copy numbers detected under standard culture conditions to those detected under inactivated conditions:

$${{{\rm{Replication\; coefficient}}}}=\frac{{{{\rm{Viral\; copies}}}}({{{\rm{standard\; culture}}}})}{{{{\rm{Viral\; copies}}}}({{{\rm{inactivated\; culture}}}})}$$

A replication coefficient greater than 2 was considered indicative of productive viral replication in the tissue. The PCR primers used were as follows: OVM-NSP2 forward primer: 5′-GGGATTCACTACACCTGCTTAGAC-3′; OVM-NSP2 reverse primer: 5′-GCTGACTCTGTCTGCGTAACC-3′; and OVM-NSP2 probe: 5′-CTCTCATCAGCAGCGAGCCTCCT-3′.

### Animal models

For tumor inoculation, cells from all the lines were harvested via 0.05% trypsin, washed sequentially with complete medium and phosphate-buffered saline (PBS), and centrifuged. The cell pellets were then resuspended in PBS for subcutaneous injection (200 µL injection volume) or in a 1:1 PBS/methylcellulose mixture for intracranial injection (5 µL injection volume). 1 × 10^5^ GL261, 1 × 10^4^ GL261-Luc, 1 × 10^4^ CT2A, and 1 × 10^5^ GL261-OVA were injected intracranially. A total of 1 × 10^6^ U-87 MG were injected subcutaneously. At 5–9 days post-inoculation, the mice were intravenously administered 300 µL of OVM (2 × 10⁷ CCID_50_/mL) via the tail vein.

For the splenectomy model, the mice were anesthetized via an intraperitoneal injection of pentobarbital sodium (50 mg/kg, 0.75% solution). The surgical site over the spleen was shaved and disinfected with iodophor. After the skin and fascia were incised, the spleen was exposed. Splenic vessels were ligated, and the spleen was excised following transection of its vascular connections and surrounding tissues. The wound was sutured and disinfected, and the animals were monitored postoperatively until they fully recovered from anesthesia.

### In vivo imaging

The mice received an intraperitoneal injection of D-Luciferin potassium salt (150 mg/kg) (Absin, abs42075819) for bioluminescence substrate delivery. Prior to imaging, anesthesia was induced via the intraperitoneal administration of pentobarbital sodium (50 mg/kg, 0.75% solution). Bioluminescent signals were captured via an IVIS Spectrum system (PerkinElmer). The tumor burden was quantified by measuring the total flux (photons/second) within standardized regions of interest.

### Serum IL-10 and TGF-β1 detection

The concentrations of IL-10 and TGF-β1 in the serum of glioma-bearing mice were detected via ELISA, as previously described in the literature [50]. A Mouse IL-10 ELISA Kit (MULTI SCIENCES, EK210) and a Mouse TGF-β1 ELISA Kit (MULTI SCIENCES, EK981) were used for detection, following the manufacturers’ instructions.

### In vivo antibody administration

For the B-cell depletion experiments, the mice received intraperitoneal injections of 500 µg of anti-mouse CD19 (clone 1D3; Selleck, A2149) 1 day prior to tumor inoculation. Additional intraperitoneal injections of 250 µg of anti-mouse CD19 were administered on day 2 and day 7 posttumor inoculation. For the anti-PD-1/anti-PD-L1 antibody administration experiments, the mice received intraperitoneal injections of 250 µg of anti-mouse PD-1 antibody (clone RMP1-14; Selleck, A2122) or 250 µg of the rat IgG2a isotype control-InVivo (clone 2A3; Selleck, A2123) on days 5, 7, and 9 after tumor inoculation. For the anti-PD-L1 treatment group, the mice were intraperitoneally injected with 250 µg of anti-mouse PD-L1 antibody (clone RMP1-14; Selleck, A2115) or 250 µg of the rat IgG2b isotype control-InVivo (clone LTF-2; Selleck, A2116) at the same time points (days 5, 7, and 9 posttumor inoculation).

### B cells, MZ B cells, Bst2^+^ B cells, CD8^+^ T cells, and DC isolation

Single-cell suspensions from mouse spleens were generated by mechanical dissociation through 40-µm cell strainers (BIOFIL, CSS-010-040), followed by red blood cell lysis (TIANGEN, RT122-02).

For total B-cell isolation, the EasySep™ Mouse B-Cell Isolation Kit (STEMCELL Technologies, 19854) was used. The single-cell suspension was adjusted to 1 × 10^8^ cells/mL (0.25–2 mL), and an FcR blocker (20 μL per mL of sample) was then added to the suspension. The isolation cocktail (50 μL per mL of sample) was subsequently added, and the mixture was incubated at room temperature for 10 min. RapidSpheres™ were vortexed for 30 s, after which they were added to the sample (50 μL per mL of sample), and the mixture was incubated at room temperature for 2.5 min. The sample was subsequently brought to 2.5 mL with the recommended medium, and the lidless tube was placed into the kit-matched magnet (EasySep™, Catalog #18000) and incubated at room temperature for 2.5 min. Finally, the magnet and tube were inverted together, and the enriched B-cell suspension was poured into a new tube. The purity of the isolated total B cells was verified via flow cytometry, and the isolation efficiency exceeded 96% (Supplementary Fig. S8A).

For MZ B-cell enrichment (EasySep™ Mouse FITC Positive Selection Kit II, STEMCELL Technologies, 17668), purified B cells were adjusted to 1 × 10^8^ cells/mL (0.1–2.5 mL) and transferred to the required tube. The FcR blocker (10 μL/mL sample) and Cr2-FITC antibody (1 μg/mL sample) were sequentially added, and the mixture was incubated for 15 min at room temperature. The selection cocktail (100 μL/mL sample) was added for 15 min. RapidSpheres™ (30 s) were vortexed and added (50 μL/mL sample) for 10 min of incubation. The sample was brought to 2.5 mL, and then the lidless tube was placed in the magnet for 5 min. The supernatant was discarded by inverting the assembly, and this separation process was repeated twice. The retained cells were MZ B cells. The purity of the enriched MZ B cells was verified via flow cytometry, and the isolation efficiency exceeded 93% (Supplementary Fig. S8B).

For Bst2^+^ B-cell isolation, purified MZ B cells were cultured in vitro with OVM (MOI of 10) and IL-4 (20 ng/mL) for 48 h. After incubation, the cells were harvested, washed twice with cold PBS containing 2% FBS, and stained with Bst2-APC antibody (eBioscience, 17-3172-82) for 30 min at 4 °C in the dark. The cells were then sorted to isolate Bst2^+^ and Bst2^−^ B-cell subsets via a Beckman mFlo flow cytometer.

For CD8⁺ T-cell isolation, the density of the splenic cell suspensions was adjusted to 1 × 10^8^ cells/mL. Biotin-Ab mixture (20 μL/mL sample) was added, and the mixture was incubated at 4 °C for 10 min. Streptavidin beads (200 μL/mL sample, vortexed to resuspend prior to use) were then added, followed by incubation at 4 °C for 10 min. Sorting buffer (2.5 mL) was added to the lidless tube, which was placed in the magnet for 5 min. The tube and magnet were inverted together, and the collected cell suspensions were purified from CD8^+^ T cells. The purity of the isolated CD8^+^ T cells was verified via flow cytometry, and the isolation efficiency exceeded 92% (Supplementary Fig. S8C).

For DC isolation (EasySep™ Mouse CD11c Positive Selection Kit, STEMCELL Technologies, 18780), the splenic single-cell suspension was adjusted to 1 × 10^8^ cells/mL (0.5–2 mL), and an FcR blocker (60 μL per mL of sample) was added to the suspension. A selection cocktail (50 μL per mL of sample mixed from equal volumes of Component A and Component B) was prepared, incubated at room temperature for 5 min, and then added to the sample for 5 min of room temperature incubation. RapidSpheres™ were vortexed for 30 s and added (40 μL per mL of sample), followed by 3 min of room temperature incubation. The sample was topped to 2.5 mL with the recommended medium, and the lidless tube was placed into a kit-matched magnet (EasySep™, Catalog #18000) for 3 min. The magnet and tube were inverted to discard the supernatant, and this separation process was repeated three more times (4 total 3-min separations). The retained cells were resuspended in the desired medium to yield purified CD11c^+^ cells. The purity of the isolated DCs was verified via flow cytometry, and the isolation efficiency exceeded 90% (Supplementary Fig. S8D).

### In vitro coculture

B cells were isolated from the spleens of glioma-bearing mice treated with either vehicle or OVM, whereas CD8^+^ T cells were isolated from vehicle-treated glioma-bearing mice. B cells and CD8^+^ T cells were cocultured in RPMI-1640 medium supplemented with 10% FBS at B:T ratios of 1:1, 5:1, or 10:1.

For transwell assays, B cells and CD8^+^ T cells were cocultured at a B:T ratio of 5:1 under two conditions: direct cell‒cell contact or physical separation by a transwell insert (BIOFIL, TCS016006).

For cell contact visualization, B cells were labeled with Cy5 (MCE, HY-D0819), and CD8^+^ T cells were labeled with CFSE (Invitrogen, C34554) prior to coculture at a B:T ratio of 5:1. After 24 h, dynamic live-cell imaging was performed via a fully motorized fluorescence microscope equipped with a live-cell incubation system (Nikon ECLIPSE Ti2).

For inhibitor studies targeting the immunological synapse, proteasome, lysosome, or ER-associated degradation (ERAD) pathways, B cells were pretreated for 24 h with vehicle (DMSO), the ICAM1–LFA interaction inhibitor A-286982 (Selleck, S3408) (1 µM), the proteasome inhibitor lactacystin (MCE, HY-16594) (2.5 µg/mL), the lysosome inhibitor cathepsin inhibitor I (TargetMol, 225120--65--0) (10 µM), or the ERAD inhibitor Eeyarestatin I (TargetMol, 412960--54--4) (3 µM). The cells were then cocultured with CD8^+^ T cells at a 5:1 ratio.

For MHC-I blockade, B cells and CD8^+^ T cells were cocultured at a 5:1 ratio in the presence of InVivoMAb anti-mouse MHC Class I (H-2) (clone M1/42.3.9.8; Bioxcell, BE0077) (100 µg/mL).

For the OVA uptake assay, B cells were isolated from the spleens of GL261-OVA-bearing mice and coincubated with ovalbumin-Cy5 (MCE, HY-NP055) (10 µg/mL) for 24 h, after which OVA-Cy5 uptake by B cells was assessed via flow cytometry.

### Flow cytometry

For apoptosis detection following OVM infection, glioma cells were harvested with EDTA-free trypsin and centrifuged at 300 × *g* for 5 min (4 °C), and the supernatant was discarded. The cells were washed once with precooled PBS, recentrifuged, and resuspended in 100 μL of 1× Annexin V Binding Buffer (from the Annexin V-APC/PI Apoptosis Kit, Elabscience, E-CK-A217) to a concentration of 1 × 10^6^ cells/mL. Then, 2.5 μL of Annexin V-APC and 2.5 μL of PI reagent (provided in the kit) were added to the cell suspension, which was vortexed gently and incubated at room temperature in the dark for 20 min before being diluted with 400 μL of precooled 1× Annexin V binding buffer for immediate analysis.

For cell sample preparation, single-cell suspensions from tumor tissues were prepared via the Tumor Dissociation Kit (Miltenyi Biotec, 130-096-730) according to the manufacturer’s protocol. Splenocytes were isolated as previously described, and single-cell suspensions derived from tumors, spleens, and PBMCs were subjected to red blood cell (RBC) lysis buffer treatment to remove erythrocytes.

Surface marker staining was performed as follows: first, the cells were stained with Fixable Viability Stain 780 (BD Biosciences, 565388) at a 1:1000 dilution (0.1 μL per 100 μL of cell suspension) with immediate vortexing and incubated for 15 min at room temperature in the dark to exclude dead cells. The cells were subsequently incubated with a CD16/CD32 monoclonal antibody (eBioscience, 14-0161-81) at a final concentration of 0.5 μg per test to block nonspecific Fc-mediated binding. Finally, the cells were stained with a panel of fluorochrome-conjugated surface antibodies and isotype controls at the manufacturer-recommended concentrations, with detailed information on all the antibodies and isotype controls provided in Supplementary Table 1. Staining was performed at 4 °C in the dark for 30 min, followed by two washes with precooled PBS.

For OVA-specific T-cell detection, after surface marker staining, the cells were resuspended in 50 μL of precooled PBS, and 1 μL of SIINFEKL-MHC tetramer-PE (BetterGen, BTG14028) was added to the 50 μL staining system. The mixture was incubated at 4 °C for 1 h in the dark and then washed twice with precooled PBS.

Intracellular staining was conducted via the Intracellular Fixation & Permeabilization Buffer Set (eBioscience, 88-8824-00): following surface staining and the final PBS wash, the supernatant was discarded, and the cell pellet was vortexed briefly to completely dissociate the cell clumps, with approximately 100 μL of residual volume retained. One hundred microliters of Intracellular Fixation Buffer was added to the cell pellet, followed by brief vortexing to mix thoroughly, and the mixture was incubated at room temperature for 30 min in the dark. After fixation, 2 mL of 1× permeabilization buffer was added, and the cells were centrifuged at 500 × *g* for 5 min at room temperature before the supernatant was discarded; this wash step was repeated once. The cell pellet was resuspended in 100 μL of 1× permeabilization buffer, and intracellular antibodies were added, followed by incubation at room temperature for 30 min in the dark. After staining, 2 mL of 1× permeabilization buffer was added, and the cells were centrifuged at 500 × *g* for 5 min at room temperature, after which the supernatant was discarded.

For the T-cell proliferation assay, CD8^+^ T cells were stained with 1 μM CFSE (Invitrogen, C34554) or 1 μM CellTrace Violet (Invitrogen, C34571) according to the manufacturer’s protocol: the cells were incubated for 20 min at room temperature or 37 °C protected from light, five times the original staining volume of culture medium (containing at least 1% protein) was added to quench free dye, and the mixture was incubated for an additional 5 min. The cells were pelleted by centrifugation and resuspended in fresh prewarmed complete culture medium, and the isolated B cells and labeled CD8^+^ T cells (including OT-1 CD8^+^ T cells) were cocultured at a 5:1 ratio (B:CD8^+^ T) in 48-well plates with positive control plates precoated with 3 µg/mL anti-CD3 (clone 145-2C11; BioLegend) and 5 µg/mL anti-CD28 (clone 37.51; BioLegend) antibodies.

For the T-cell function assay, positive control wells were treated with PMA (MCE, HY-18739) and ionomycin (MCE, HY-13434), and all experimental groups were supplemented with β-mercaptoethanol and brefeldin A (MCE, HY-16592). The cells were subjected to surface marker staining followed by the intracellular staining protocol described above.

All flow cytometric analyses were performed on a CytoFLEX flow cytometer (Beckman Coulter, USA). The data were analyzed with CytExpert software (Version 2.4.0.28), and histograms were specifically analyzed via FlowJo software (FlowJo, USA; Version 10.8.1). The detailed gating strategies are provided in Supplementary Fig. S9.

### Adoptive transfer experiments

Splenic B cells were isolated from GL261-OVA-bearing C57BL/6J wild-type mice to obtain vehicle-treated B cells. MZ B cells were further purified from the spleens of GL261-OVA-bearing C57BL/6J wild-type mice and treated with OVM in vitro to generate Bst2^+^ and Bst2^−^ B-cell subsets. Orthotopic GL261-OVA models were established in Ighm-KO mice, and 5 days later, the mice were adoptively transferred with vehicle B cells, Bst2^+^ B cells, Bst2^−^ B cells, or vehicle control.

### Multiplex immunofluorescence

Spleens were fixed in 4% paraformaldehyde (PFA), embedded in paraffin, and sectioned. The paraffin-embedded sections were incubated at 60 °C for 1 h, followed by deparaffinization and antigen retrieval. For antibody labeling, 0.5 µg of primary antibody was mixed with 1 µL of FlexLinker, and the volume was adjusted to 8 µL with FlexBuffer. The mixture was gently mixed and incubated for 5 min at room temperature in the dark. Subsequently, 2 µL of FlexQuencher was added, followed by gentle mixing and incubation for an additional 5 min under the same conditions. The final volume was adjusted to 50–100 µL with TBS. A mixture of multiple FlexAble-labeled antibodies was applied to the sections, which were subsequently incubated overnight at 4 °C in the dark. After incubation, the sections were mounted with Antifade Mounting Medium with DAPI (Beyotime Biotechnology, P0131) and scanned via a Digital Pathology Slide Scanner (KFBIO, KF-PRO-020). This multiplex immunofluorescence approach allows simultaneous detection of multiple antigens within the same tissue section by conjugating distinct fluorophores to different antibodies, enabling spatial mapping of cell populations and protein expression. The antibodies and reagents used were as follows: CD21/CD35 recombinant antibody (Proteintech, 84565-2-RR), CD19 polyclonal antibody (Proteintech, 27949-1-AP), BST2 polyclonal antibody (Proteintech, 30118-1-AP), FlexAble 2.0 Cora Lite® Plus 488 Antibody Labeling Kit for Rabbit IgG (Proteintech, KFA501), FlexAble 2.0 Cora Lite® Plus 594 Antibody Labeling Kit for Rabbit IgG (Proteintech, KFA509), and FlexAble 2.0 Cora Lite® Plus 555 Antibody Labeling Kit for Rabbit IgG (Proteintech, KFA502).

### Single-cell RNA sequencing

Splenocytes were harvested from glioma-bearing mice receiving either vehicle or OVM treatment (*n* = 3 per group). Single-cell suspensions were assessed for quality and counted, with only those exhibiting >80% viability retained. The cells were washed, resuspended, adjusted to a final concentration of 700–1200 cells/µL, and then submitted to Majorbio Biopharm Technology Co., Ltd. (Shanghai, China) for single-cell RNA sequencing. Single-cell capture and barcoding were performed on the 10x Genomics Chromium platform, in which gel beads coated with unique molecular identifiers (UMIs) and cell barcodes were loaded at near saturation to ensure one bead per gel bead-in-emulsion (GEM). Within each GEM, the cells were lysed, and the mRNA was reverse transcribed to capture transcript information with cell-specific barcodes. Following GEM generation, the emulsion was broken, and the cDNA was purified, enriched, and amplified to construct 3′ gene expression libraries according to the manufacturer’s protocol (10x Genomics). Library sequencing was conducted on the Illumina XPlus platform, with initial sequencing and bioinformatic processing performed on Majorbio Co., Ltd.’s proprietary analysis platform (Shanghai, China).

The raw sequencing data were processed via CellRanger (v7.0.1), with the *Mus musculus* genome assembly GRCm38 (mm10) used as the reference. Downstream analysis was performed via the Seurat (v4.1.1) R package. To ensure high-quality data, we applied stringent quality control (QC) criteria: cells were retained only if they contained >200 detected genes, a mitochondrial gene percentage <10%, and a log10(genes)/log10(UMI) ratio >0.8. To exclude potential doublets, the scDblFinder (v1.11.4) package was employed to identify and remove multiplet droplets. After QC, the gene expression matrices were normalized via the normalizeData function, and the top 2000 highly variable features were identified.

To integrate data from different samples and minimize batch effects, we utilized the Harmony algorithm. Principal component analysis (PCA) was first performed on the scaled data (top 50 PCs). Unsupervised clustering was then conducted via a graph-based approach: a shared nearest neighbor (SNN) graph was constructed with *k* = 50 neighbors, followed by the Louvain algorithm for modularity optimization (via FindClusters with a resolution of 0.3). The resulting clusters were visualized via both t-distributed stochastic neighbor embedding (t-SNE) and uniform manifold approximation and projection (UMAP) based on Harmony-integrated embeddings (dims 1–50, seed = 1000).

Intercellular communication was inferred and quantified via CellChat (v1.6.1). We utilized the CellChatDB mouse database to analyze ligand‒receptor interactions. Separate CellChat objects were created for the “OVM” and “Vehicle” groups. The communication probabilities were calculated, and interactions were filtered if supported by fewer than 3 cells. The overall signaling network was aggregated, and network centrality scores were computed to identify key signaling hubs. Differential interaction analysis between groups was performed via the mergeCellChat and netVisual_diff interaction functions to visualize the strength and number of increased or decreased signaling pathways in response to OVM treatment.

To investigate the developmental transitions and lineage relationships (specifically for B cells), trajectory analysis was performed via Monocle3 (v1.3.1). The Seurat-processed data and UMAP embeddings were imported into the Monocle3 environment. A principal graph was learned across the cell manifold via the learn_graph function (with use_partition = FALSE). The cells were then ordered in pseudotime via the order_cells function to calculate their progression along the developmental path.

### Statistical analysis

All the statistical analyses and graphical representations were performed via GraphPad Prism version 10.1.1, with the quantitative data conforming to normality (assessed via the Shapiro‒Wilk test) and homogeneity of variance (verified via the Brown‒Forsythe test) expressed as the mean ± standard deviation (SD); comparisons between two groups utilized unpaired two-tailed Student’s *t* tests, while multiple group comparisons employed one-way ANOVA followed by Dunnett’s post hoc test, and survival curves were analyzed via the log-rank (Mantel‒Cox) test. The 95% confidence intervals for all the statistical calculations are provided in Supplementary Table 2.

## Supplementary information

Supplementary figure1–9

Revised unprocessed images

Supplementary table1

Supplementary table2
