# Supplementary material for: Oncolytic virus M1 reinvigorates CD8+ T-cell immunity against glioblastoma through B-cell-dependent antigen cross-presentation in the spleen
Source: Cell Mol Immunol. 2026 Mar 4;23(4):349–66. doi: 10.1038/s41423-026-01396-w (PMC13035954; doi:10.1038/s41423-026-01396-w)
Supplement: Supplementary file 1 — Supplementary figure1–9 [file 41423_2026_1396_MOESM1_ESM.pdf]

Supplementary Materials

Oncolytic virus M1 reinvigorates $CD8^+$ T cell immunity against glioblastoma through B cell-dependent antigen cross-presentation in the spleen

S1

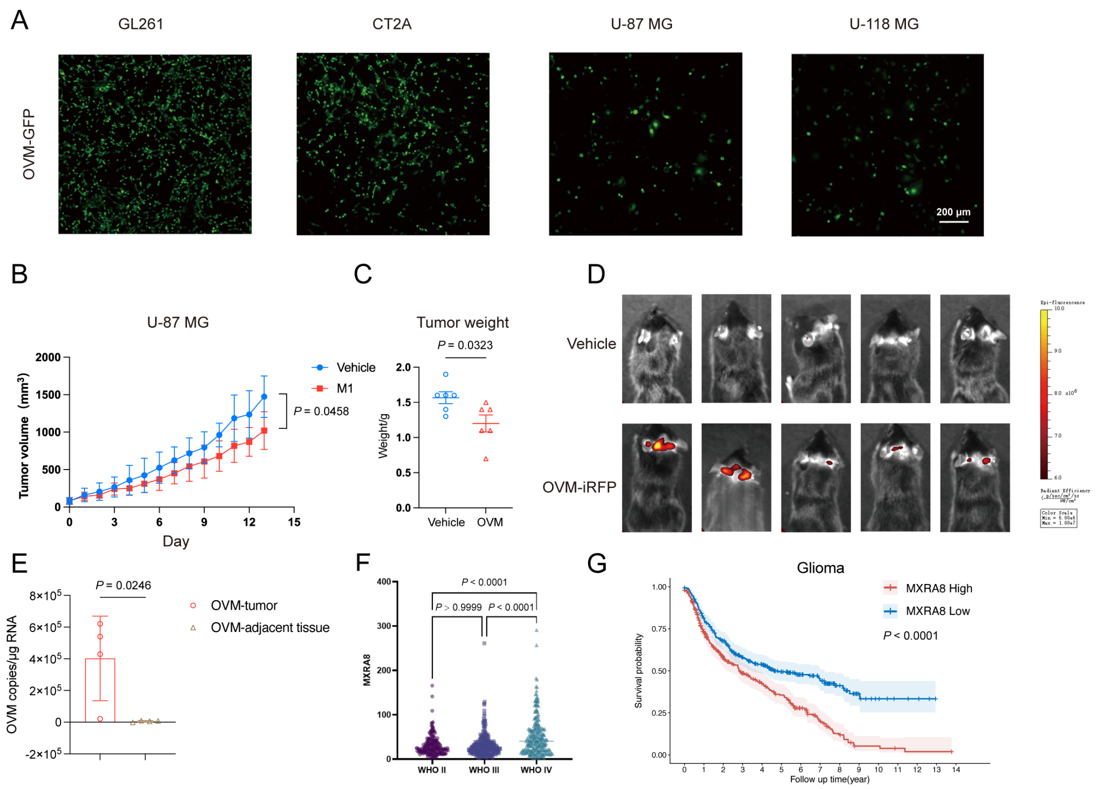

Supplementary Figure 1. Characterization of OVM infection and MXRA8 expression in glioma patients. (A) Representative fluorescence images of 4 glioma cell lines (GL261, CT2A, U-87 MG, and U-118 MG) infected with OVM-GFP (1 MOI) for 24 h. Scale bar, 200 μm. (B and C) BALB/c-nu/nu mice were subcutaneously inoculated with $1×10^6$ U-87 MG cells and subsequently treated with vehicle or OVM. Tumor growth curves showing mean tumor volume ± SEM over the indicated time course (B). Final tumor weights at the endpoint (C). (D) Representative *in vivo* fluorescence images showing red fluorescence of OVM-iRFP in GL261 tumor-bearing mice, captured using the IVIS Spectrum system. (E) qPCR analysis of OVM copies/μg RNA in tumor tissues and adjacent normal tissues from glioma orthotopic mice treated with OVM. (F) Analysis of MXRA8 expression in WHO grade II, III, and IV gliomas from the CGGA database. (G) Kaplan-Meier survival curve of glioma patients from the CGGA database, stratified by high and low MXRA8 expression.

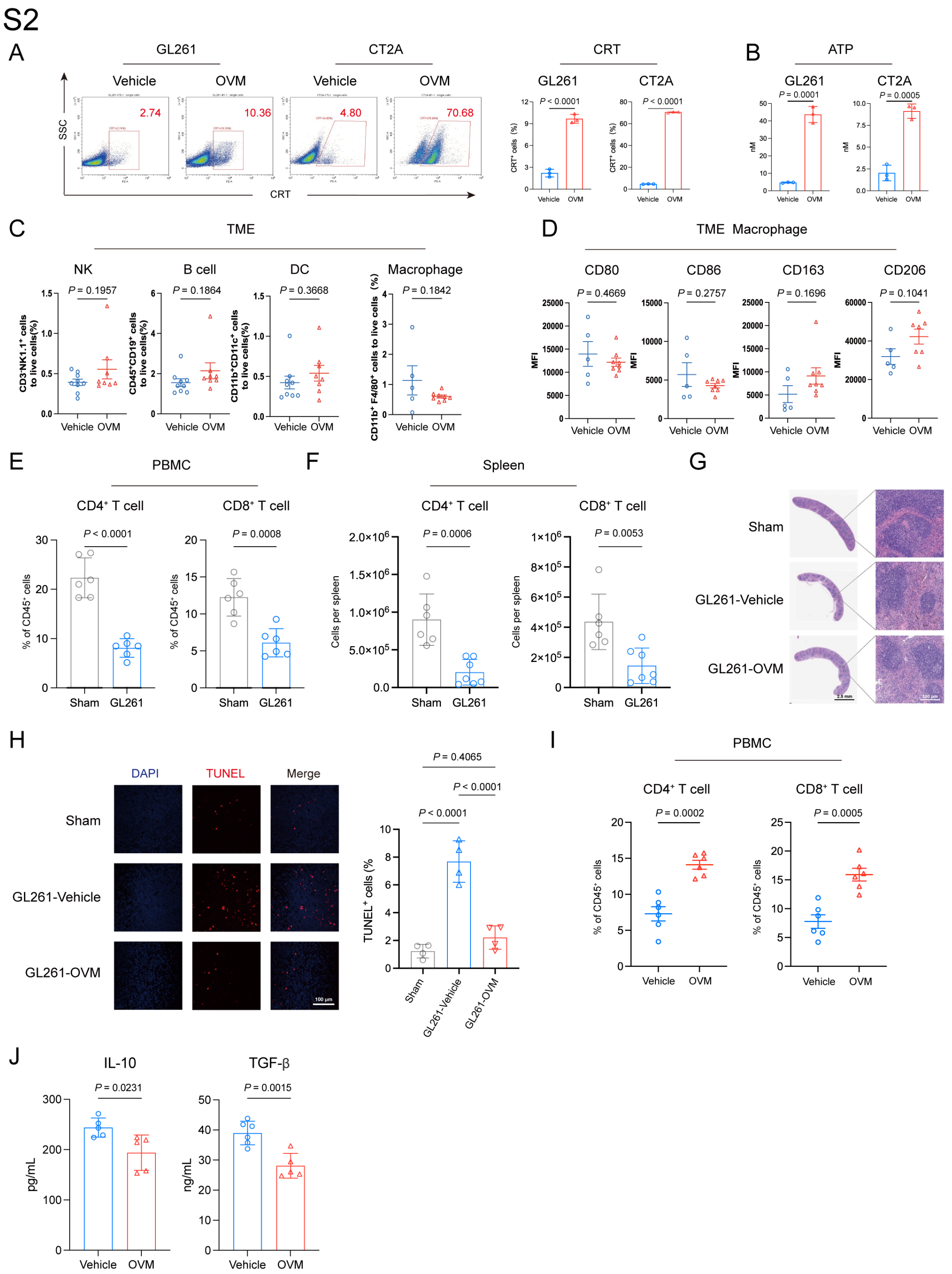

Supplementary Figure 2. Immune activation and splenic alterations in GL261 glioma models following OVM treatment. (A) Flow cytometric analysis of surface calreticulin (CRT) expression on GL261 and CT2A cells 24 h after infection with OVM at 1 MOI. (B) Measurement of extracellular ATP levels in the culture supernatants of GL261 and CT2A cells 24 h after infection with OVM at 1 MOI, assessed by ELISA. (C) Flow cytometric analysis of immune cell populations within the TME, including NK cells, B cells, dendritic cells (DCs), and macrophages. (D) Surface expression of M1 markers (CD80, CD86) and M2 markers (CD163, CD206) on macrophages within the TME, measured as mean fluorescence intensity by flow cytometry. (E and F) Quantification of

$CD4^+$ and $CD8^+$ T cell counts in PBMCs (E) and spleens (F) of GL261 tumor-bearing mice compared with sham-operated controls. (G and H) Representative H&E (G) and TUNEL (H) staining of spleens from sham-operated, GL261 tumor-bearing mice treated with vehicle, and GL261 tumor-bearing mice treated with OVM. (I) Quantification of $CD4^+$ and $CD8^+$ T cell counts in PBMCs of GL261 tumor-bearing mice. (J) ELISA quantification of IL-10 and TGF-β levels in the serum of glioma orthotopic mice treated.

S3

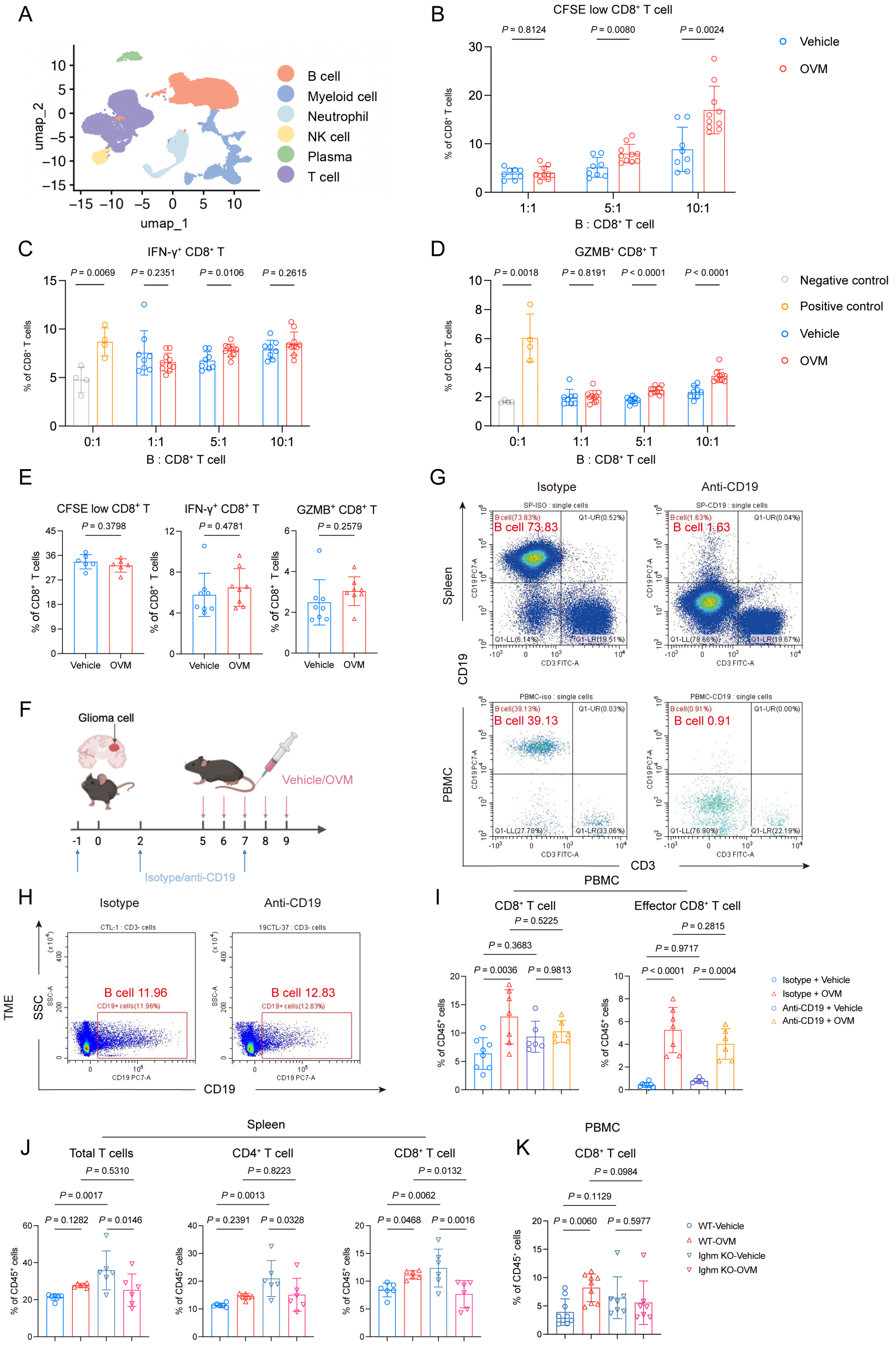

Supplementary Figure 3. Characterization of splenic immune populations and B cell-dependent CD8⁺ T cell responses in GL261 glioma models. (A) UMAP visualization of 43,926

splenic cells, analyzed using unsupervised clustering. Six immune-related cell types were identified based on canonical lineage markers: neutrophils, myeloid cells, NK cells, B cells, plasma cells, and T cells. (B–D) Splenic B cells were isolated from GL261 tumor-bearing mice treated with vehicle or OVM and co-cultured with splenic $CD8^+$ T cells from GL261 tumor-bearing mice at B:T ratios of 0:1, 1:1, 5:1, and 10:1. Proliferation rate of $CD8^+$ T cells (B), frequency of IFN-γ^+ $CD8^+$ T cells (C), and frequency of $GZMB^+$ $CD8^+$ T cells (D) were assessed by flow cytometry. (E) Splenic DCs were isolated from GL261 tumor-bearing mice treated with vehicle or OVM and co-cultured with splenic $CD8^+$ T cells from GL261 tumor-bearing mice at a DC:T ratio of 5:1. The frequency of CFSE-low $CD8^+$ T cells and IFN-γ^+ $GZMB^+$ $CD8^+$ T cells was assessed by flow cytometry. (F) Schematic of *in vivo* B cell depletion. Mice received intraperitoneal injections of 500μg anti-mouse CD19 antibody or isotype one day prior to tumor inoculation, followed by additional injections of 250μg on Days 2 and 7 post inoculation. (G and H) Validation of B cell depletion efficiency in spleen, PBMCs (G), and TME (H) of treated mice by flow cytometry. (I) Quantification of $CD8^+$ T cells and effector $CD8^+$ T cells ($CD44^+$ $CD62L^-$) in the PBMCs of GL261 tumor-bearing mice across four treatment groups (isotype + vehicle, isotype + OVM, anti-CD19 + vehicle, anti-CD19 + OVM). (J) Quantification of T cell subsets in the spleen of GL261 tumor-bearing C57BL/6J WT or Ighm-KO mice treated with vehicle or OVM. (K) Quantification of the percentage of $CD8^+$ T cells in the PBMCs of GL261 tumor-bearing C57BL/6J WT or Ighm-KO mice treated with vehicle or OVM.

S4

A

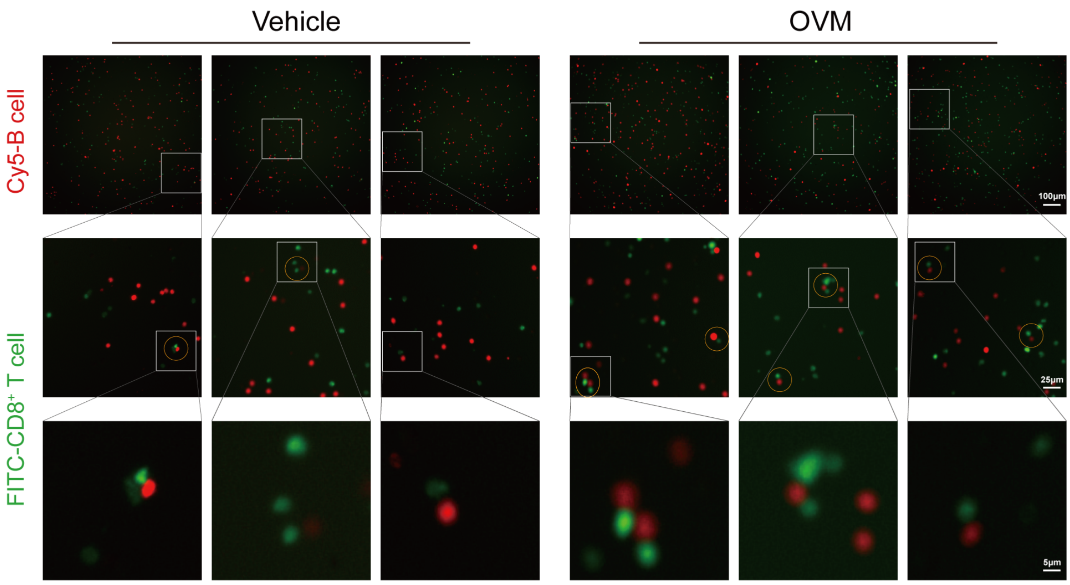

Supplementary Figure 4. Live-cell imaging of B cell–CD8^{+} T cell contacts. (A) Live-cell imaging of B cells and CD8^{+} T cells labeled with Cy5 and CFSE, respectively, to visualize their interactions. Scale bar, 100 µm, 25 µm, and 5 µm.

S5

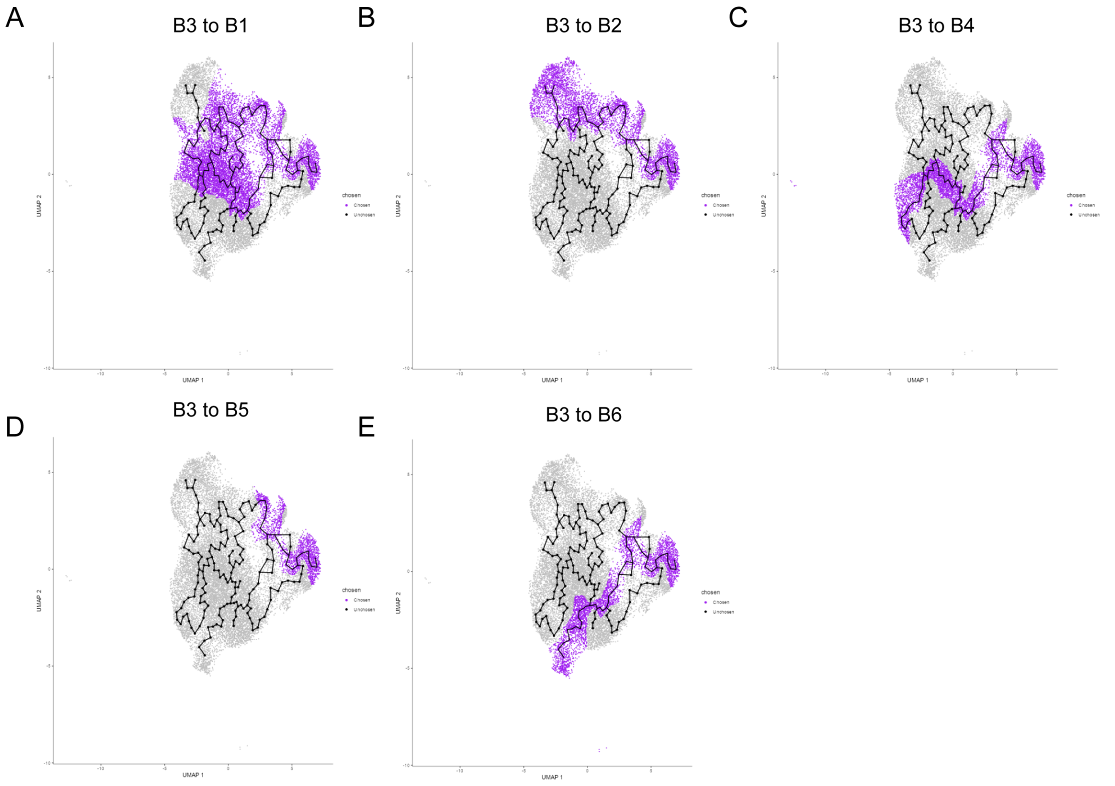

Supplementary Figure 5. Pseudotime trajectory analysis of B cell subsets. (A–E) Pseudotime trajectory plots showing the differentiation trajectories of B cell subsets: (A) Differentiation trajectory from B3 to B1; (B) Differentiation trajectory from B3 to B2; (C) Differentiation trajectory from B3 to B4; (D) Differentiation trajectory from B3 to B5; (E) Differentiation trajectory from B3 to B6.

S6

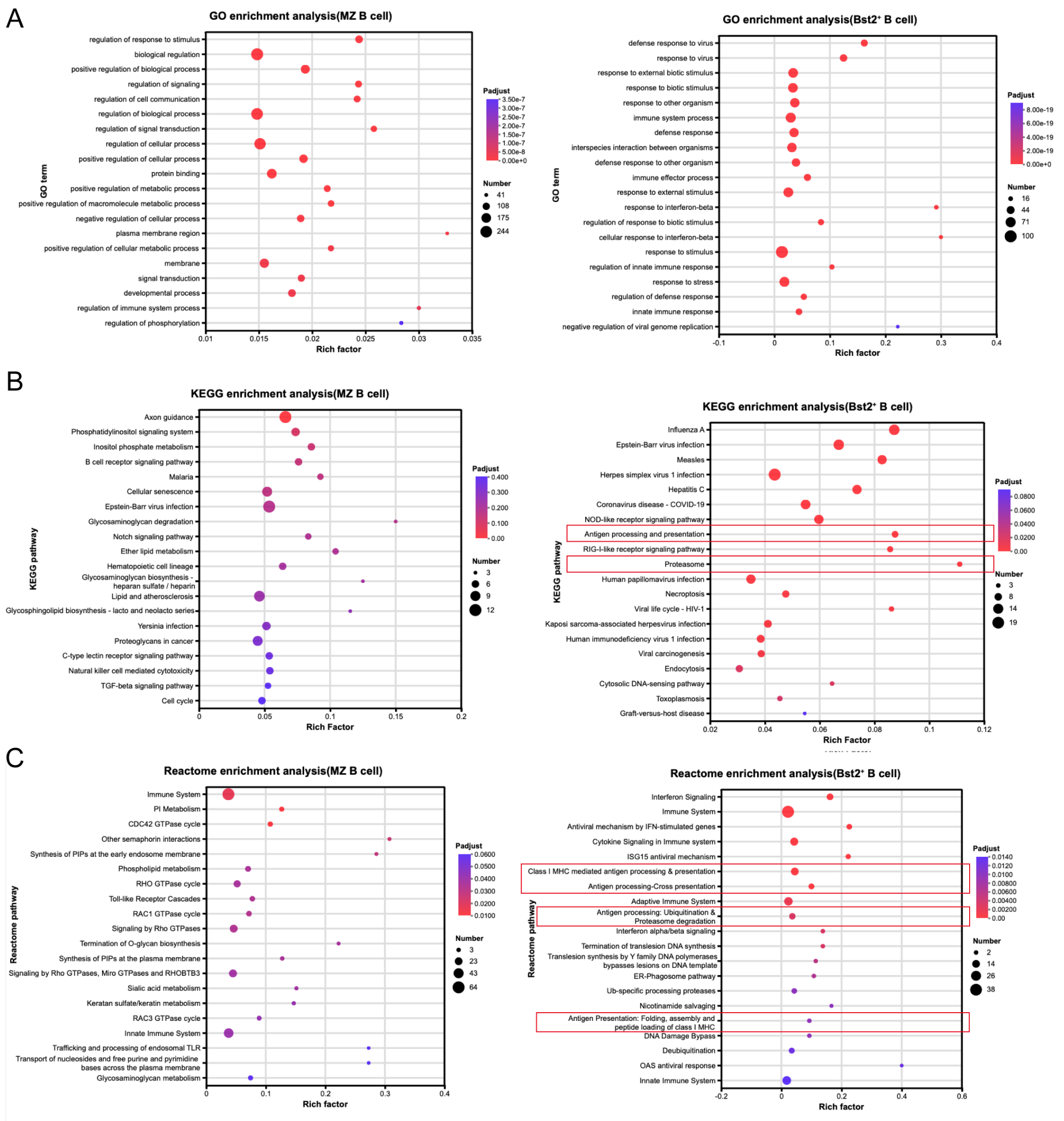

Supplementary Figure 6. Pathway enrichment analysis of signature genes in splenic MZ B cells and Bst2⁺ B cells from scRNA-seq data. (A–C) Bubble plots showing GO (A), KEGG (B), and Reactome (C) pathway enrichment of signature upregulated genes in splenic MZ B cells and Bst2^{+} B cells from GL261 tumor-bearing mice. Signature upregulated genes were defined as the marker genes of MZ B cells versus other splenic B cell subsets and those of Bst2^{+} B cells versus other splenic B cell subsets, respectively. The y-axis represents pathway names, the x-axis indicates rich factor, bubble size denotes the number of enriched genes, and color reflects adjusted *P* value.

S7

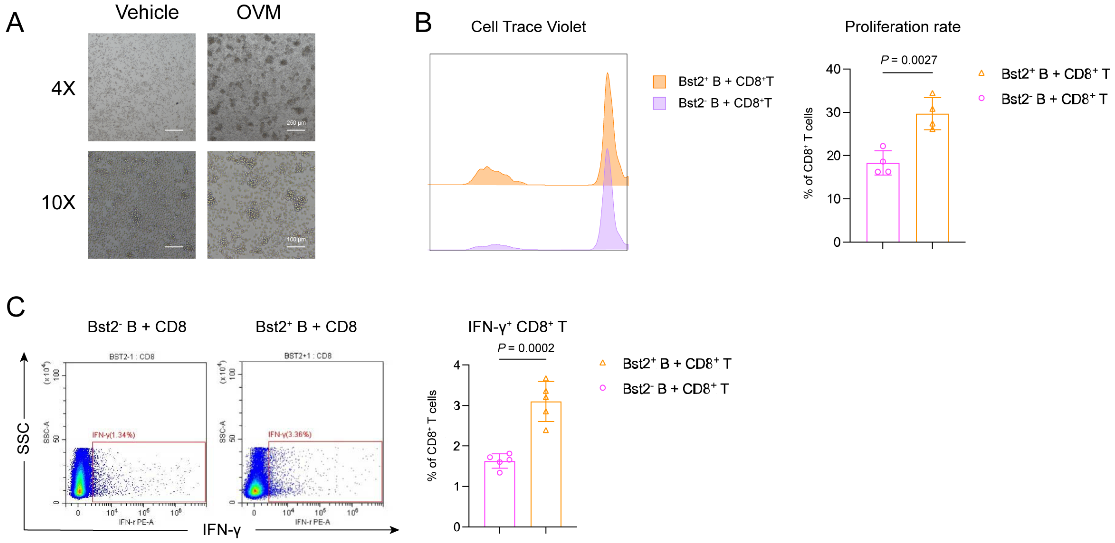

Supplementary Figure 7. OVM-induced Bst2⁺ B cells promote CD8⁺ T cell proliferation and activation. (A) Representative images of spheroid formation of MZ B cells isolated from GL261 tumor-bearing mice following *in vitro* stimulation with vehicle or OVM. Scale bar, 250 µm, 100 µm. (B and C) Splenic MZ B cells from GL261 tumor-bearing mice were stimulated *in vitro* with OVM, sorted into Bst2⁺ and Bst2⁻ populations, and co-cultured with splenic CD8⁺ T cells from GL261 tumor-bearing mice. CD8⁺ T cell proliferation was measured by Cell Trace Violet dilution (B), and activation was assessed by the frequency of IFN-γ⁺ CD8⁺ T cells (C).

S8

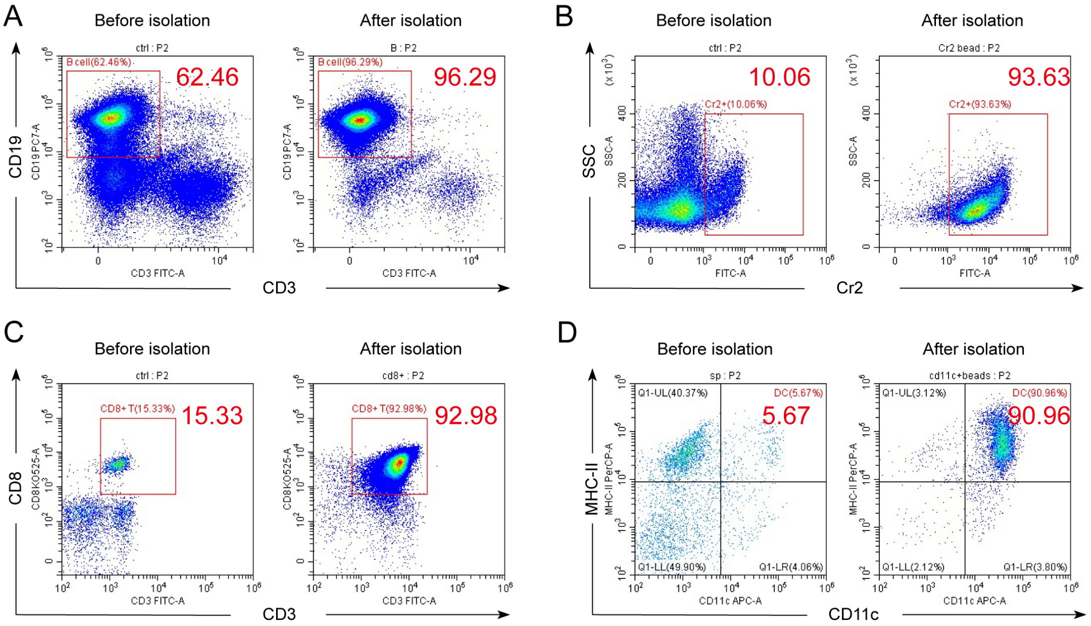

Supplementary Figure 8. Validation of cell sorting efficiency. (A) Purity validation of B cell sorting; (B) Purity validation of MZ B cell sorting; (C) Purity validation of $CD8^+$ T cell sorting. (D) Purity validation of DCs sorting. All purity analyses were performed by flow cytometry.

S9

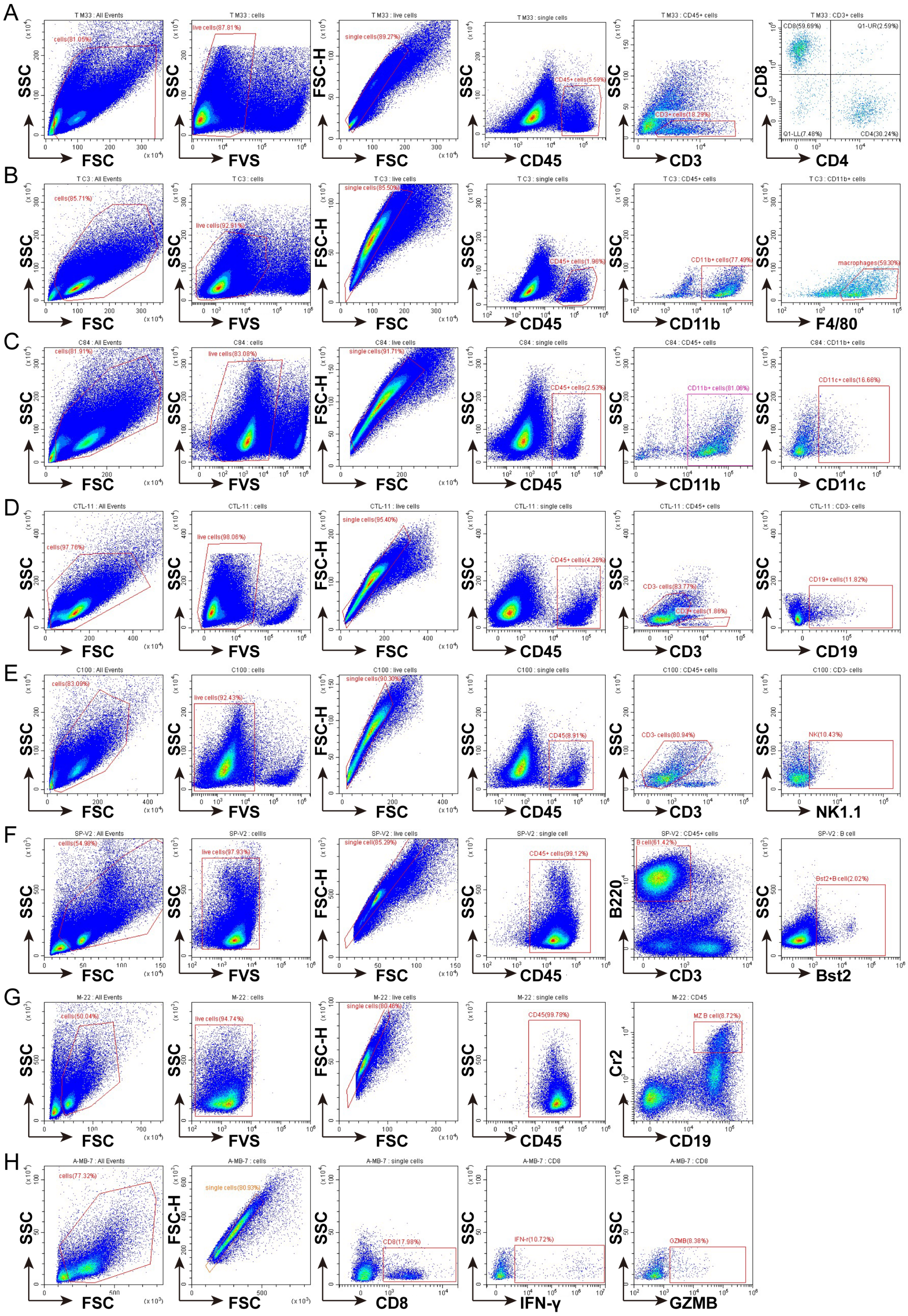

Supplementary Figure 9. Gating strategies for flow cytometric analysis of immune cell subsets. (A) Gating strategy for CD3$^+$ T cells, CD4$^+$ T cells, and CD8$^+$ T cells. Sequentially gated from all events to cells, then to viable cells (Fixable viability stain (FVS) low expression), followed by gating on CD45$^+$ cells. From CD45$^+$ cells, CD3$^+$ T cells were gated, and further subgated into CD4$^+$ T cells and CD8$^+$ T cells. (B) Gating strategy for macrophages. Gated from CD45$^+$ cells to CD11b$^+$ cells, then further gated on F4/80$^+$ cells. (C) Gating strategy for DCs. Gated from CD45$^+$ cells to CD11b$^+$

cells, then further gated on CD11c$^+$ cells. (D) Gating strategy for B cells. Gated from CD45$^+$ cells to CD3$^-$ cells, then further gated on CD19$^+$ cells. (E) Gating strategy for NK cells. Gated from CD45$^+$ cells to CD3$^-$ cells, then further gated on NK1.1$^+$ cells. (F) Gating strategy for Bst2$^+$ B cells: Gated from CD45$^+$ cells to B220$^+$CD3$^-$ cells, then further gated on Bst2$^+$ cells from the B cell population. (G) Gating strategy for marginal zone (MZ) B cells. Gated from CD45$^+$ cells to CD19$^+$Cr2$^+$ cells. (H) Gating strategy for IFN-γ^+ and GZMB$^+$CD8$^+$ T cells. Gated from CD8$^+$ T cells to IFN-γ^+ CD8$^+$ T and GZMB$^+$ CD8$^+$ T cells. (A–E) were derived from tumor microenvironment (TME) samples; (F and G) were derived from splenic cell samples; (H) was derived from co-culture system samples.
