# Supplementary material for: Oncolytic virus M1 reinvigorates CD8+ T-cell immunity against glioblastoma through B-cell-dependent antigen cross-presentation in the spleen
Source: Cell Mol Immunol. 2026 Mar 4;23(4):349–66. doi: 10.1038/s41423-026-01396-w (PMC13035954; doi:10.1038/s41423-026-01396-w)

Fig. 1E

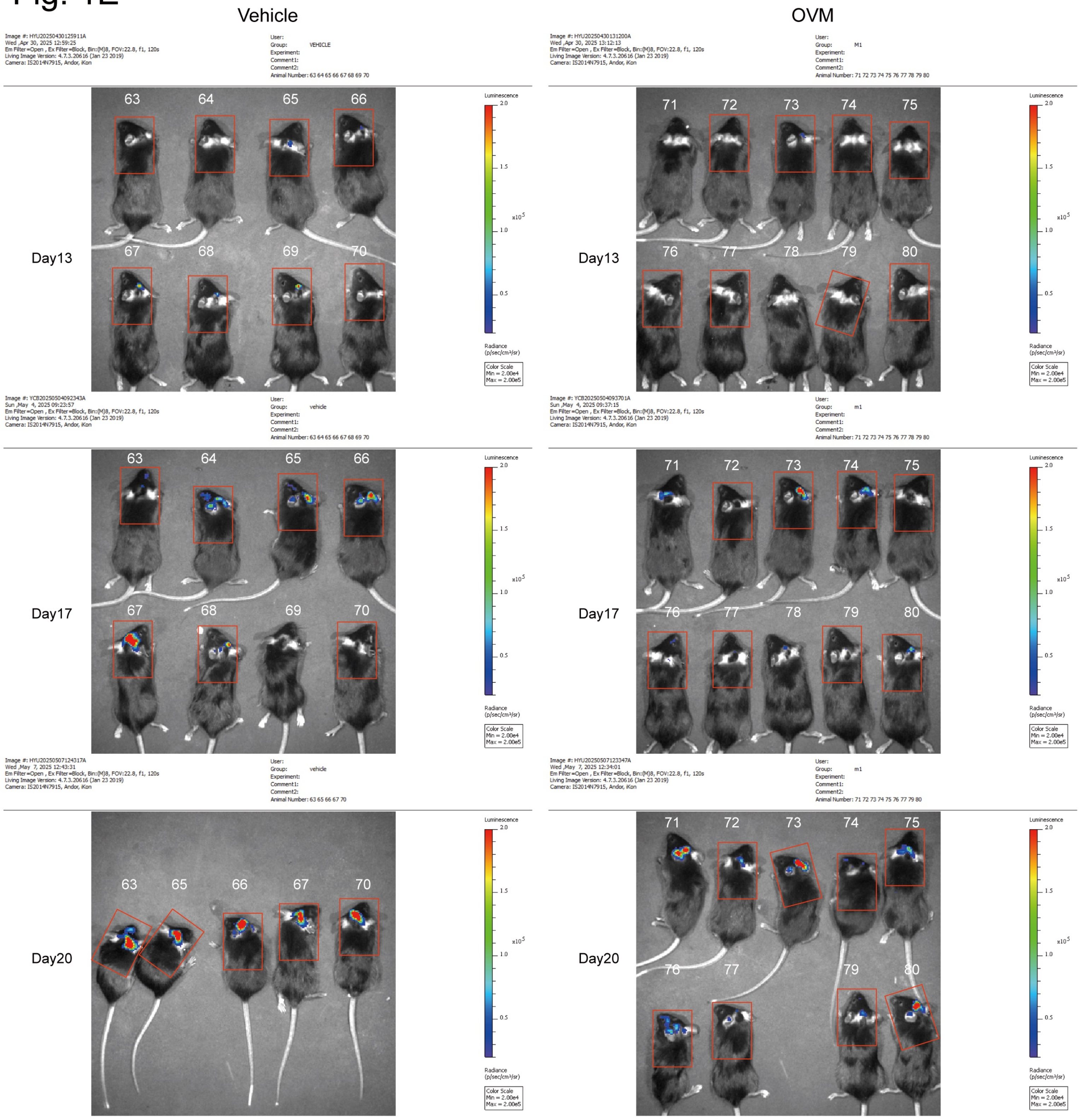

Note: On day 17, mouse #69 was moribund and tumor fluorescence imaging could not be obtained.

Note: Mouse #71 received OVM injections only on days 5 and 6 owing to poor tail vein access, resulting in three missed doses, but was still subjected to IVIS imaging. Mouse #78 did not recover from anesthesia after imaging on day 17 despite a minimal tumor burden. Both animals were excluded from the final statistical analysis.

Fig. 2D

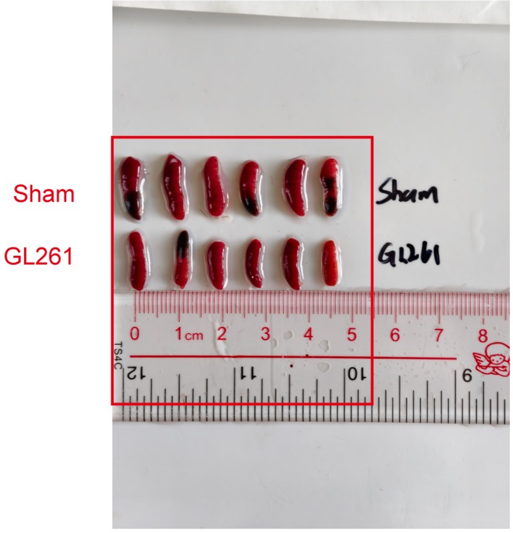

Fig. 2E

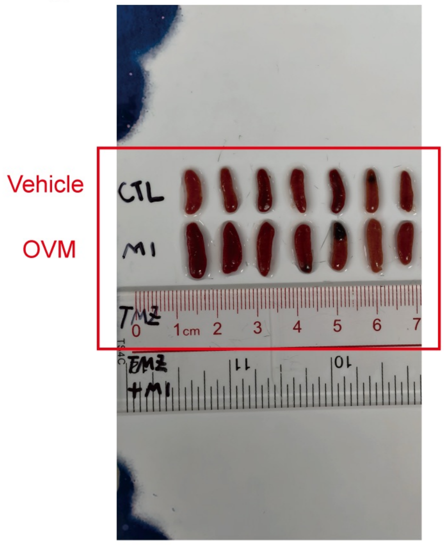

Fig. 3M

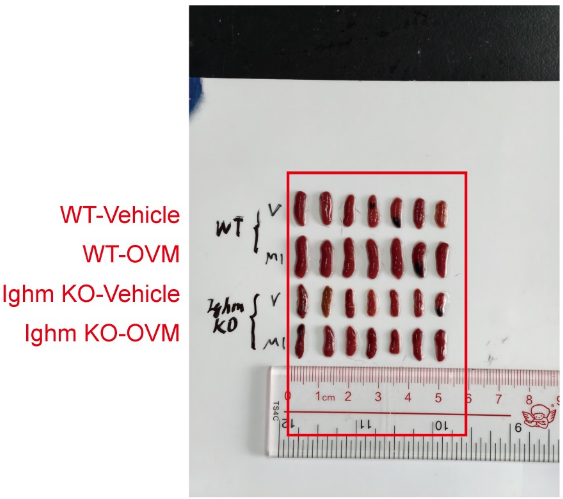

Fig. 6G

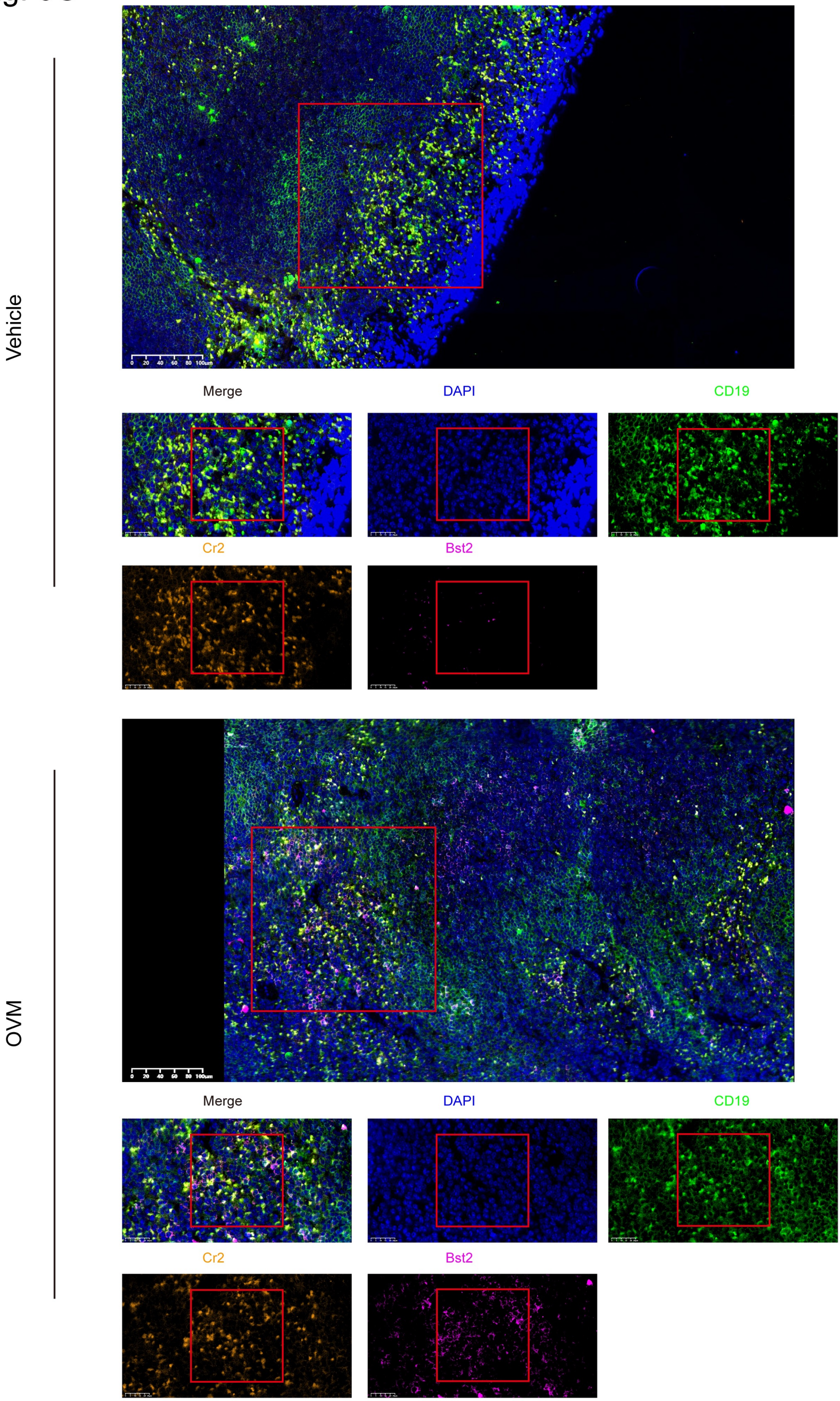
Vehicle
Merge
DAPI
CD19
Cr2
Bst2
OVM
Merge
DAPI
CD19
Cr2
Bst2

Fig. 7B

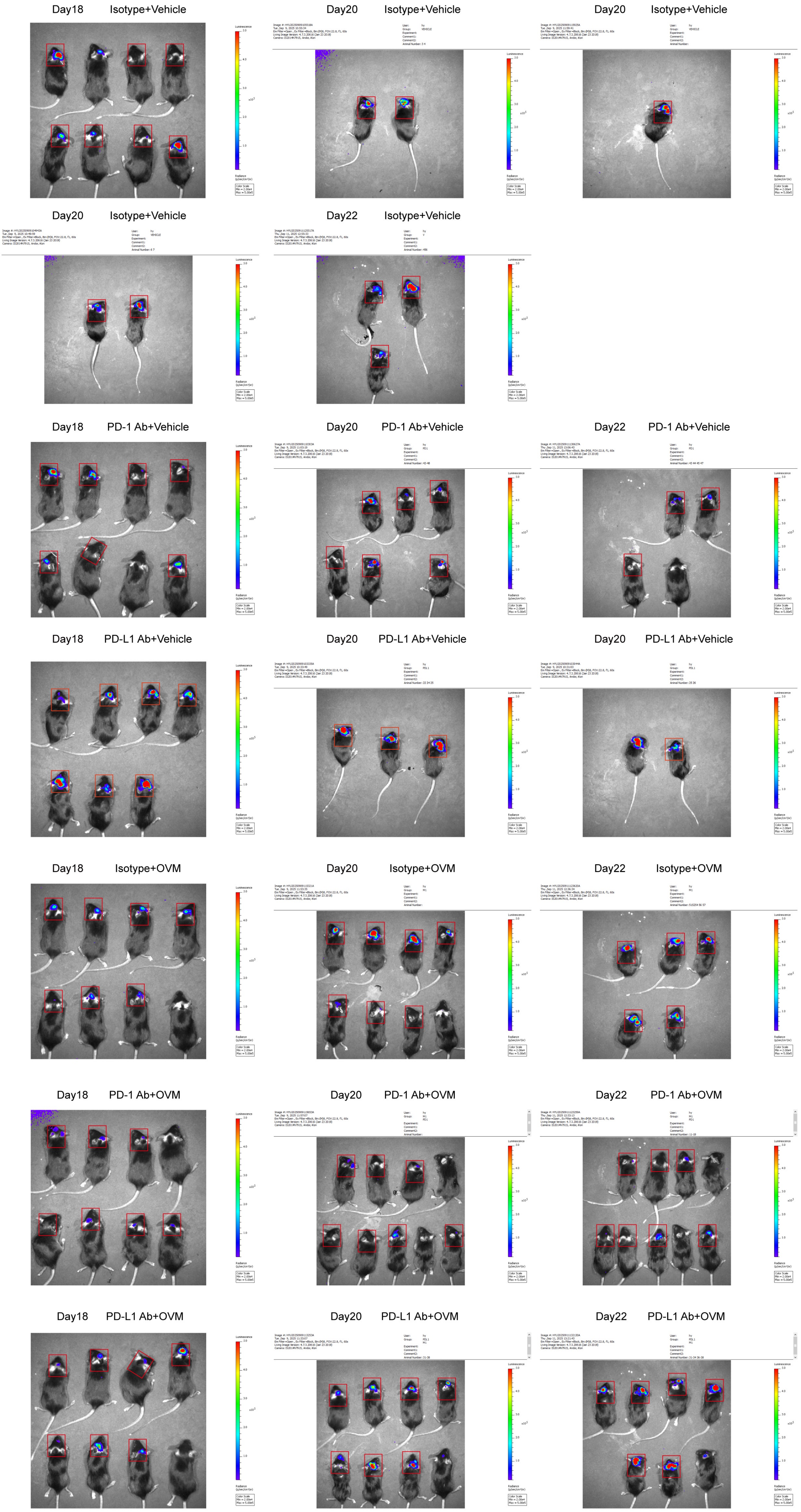
Day18 Isotype+Vehicle
Day20 Isotype+Vehicle
Day20 Isotype+Vehicle
Day20 Isotype+Vehicle
Day22 Isotype+Vehicle
Day18 PD-1 Ab+Vehicle
Day20 PD-1 Ab+Vehicle
Day22 PD-1 Ab+Vehicle
Day18 PD-L1 Ab+Vehicle
Day20 PD-L1 Ab+Vehicle
Day20 PD-L1 Ab+Vehicle
Day18 Isotype+OVM
Day20 Isotype+OVM
Day22 Isotype+OVM
Day18 PD-1 Ab+OVM
Day20 PD-1 Ab+OVM
Day22 PD-1 Ab+OVM
Day18 PD-L1 Ab+OVM
Day20 PD-L1 Ab+OVM
Day22 PD-L1 Ab+OVM

Fig. 7I

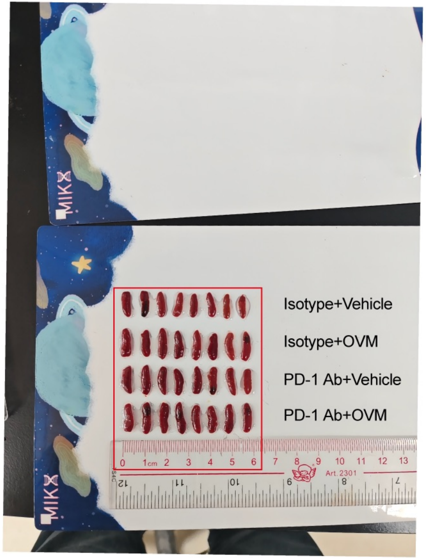

Supplementary Fig. S1A

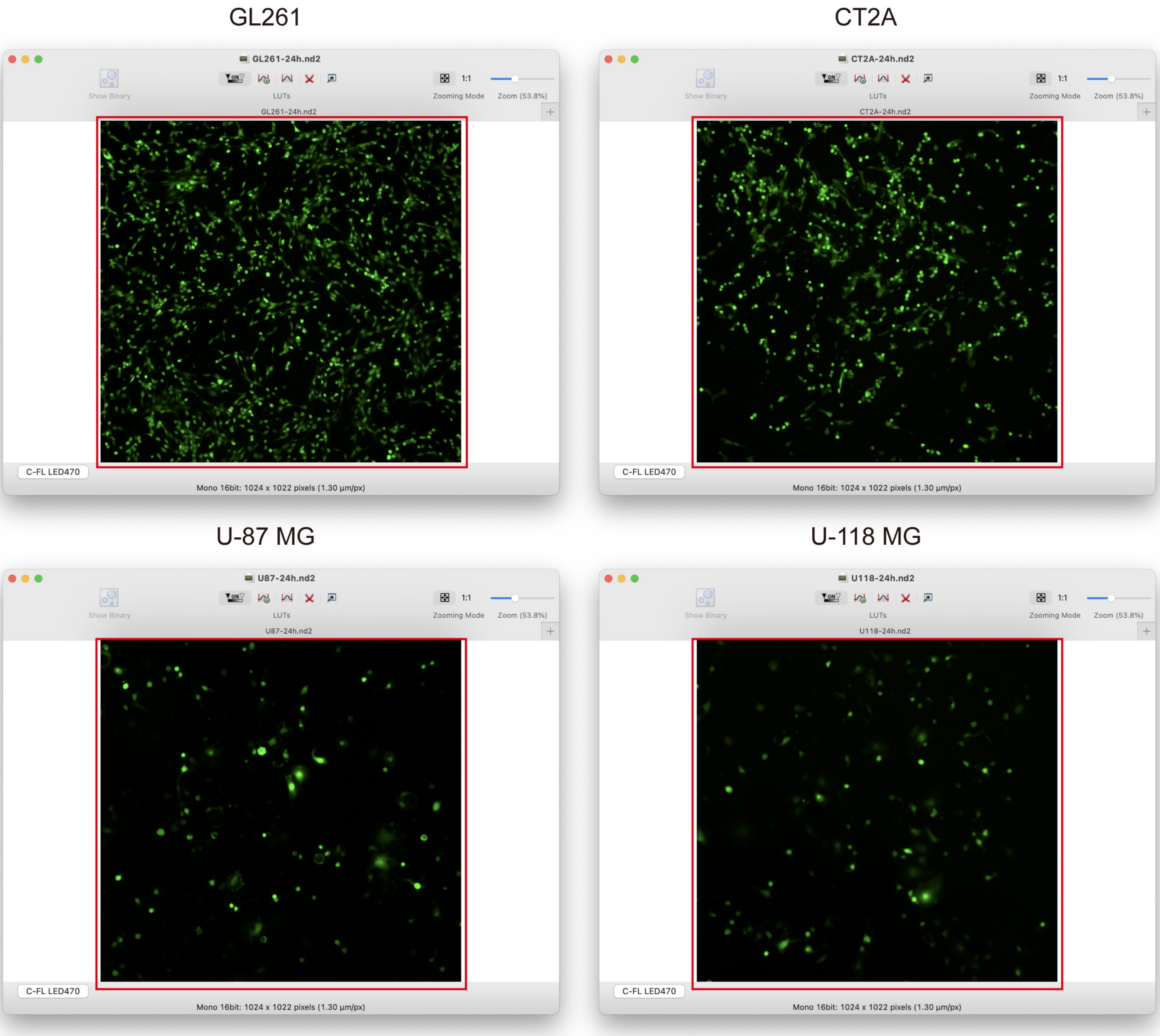

Supplementary Fig. S1D

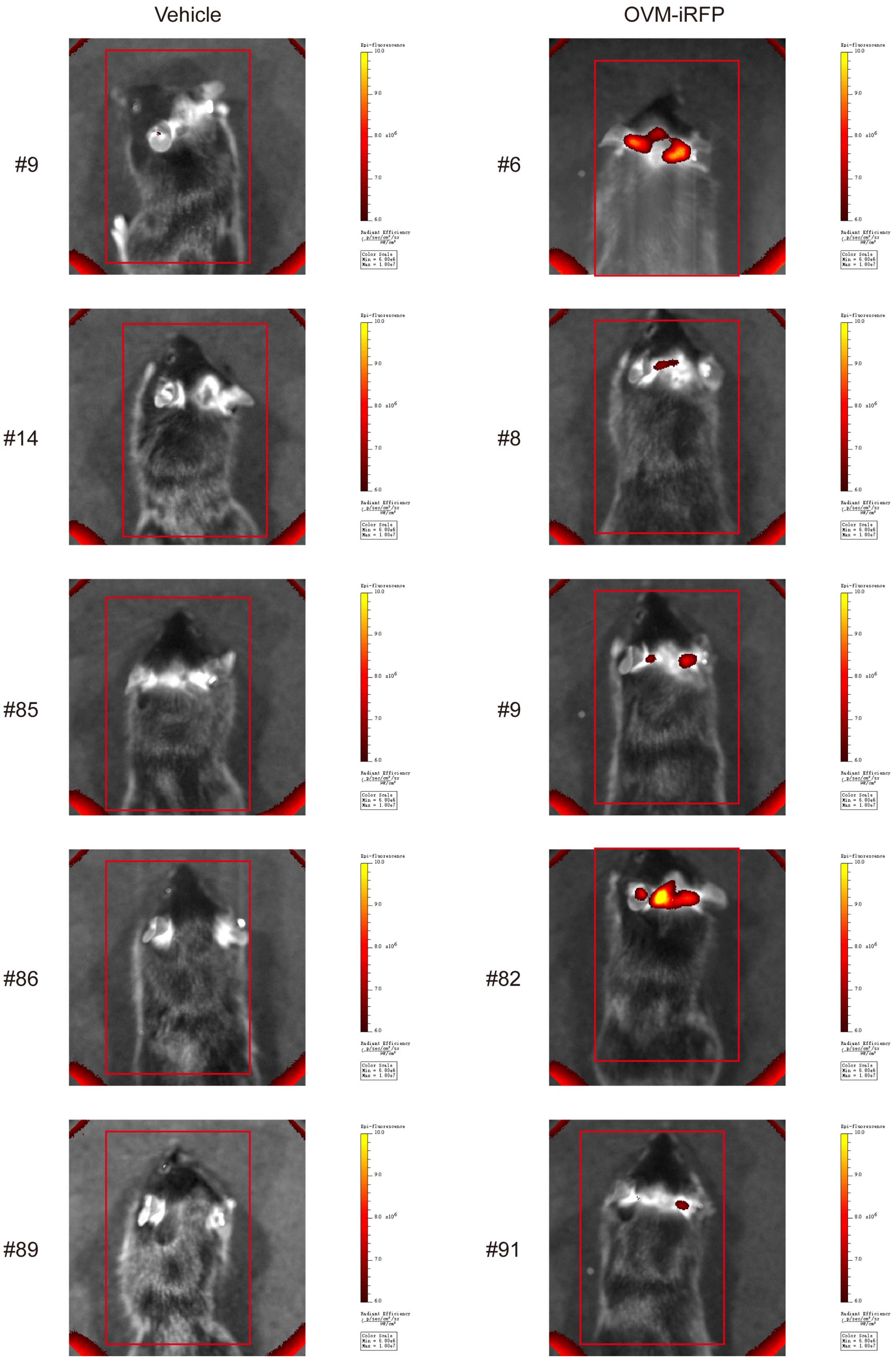

Supplementary Fig. S2G

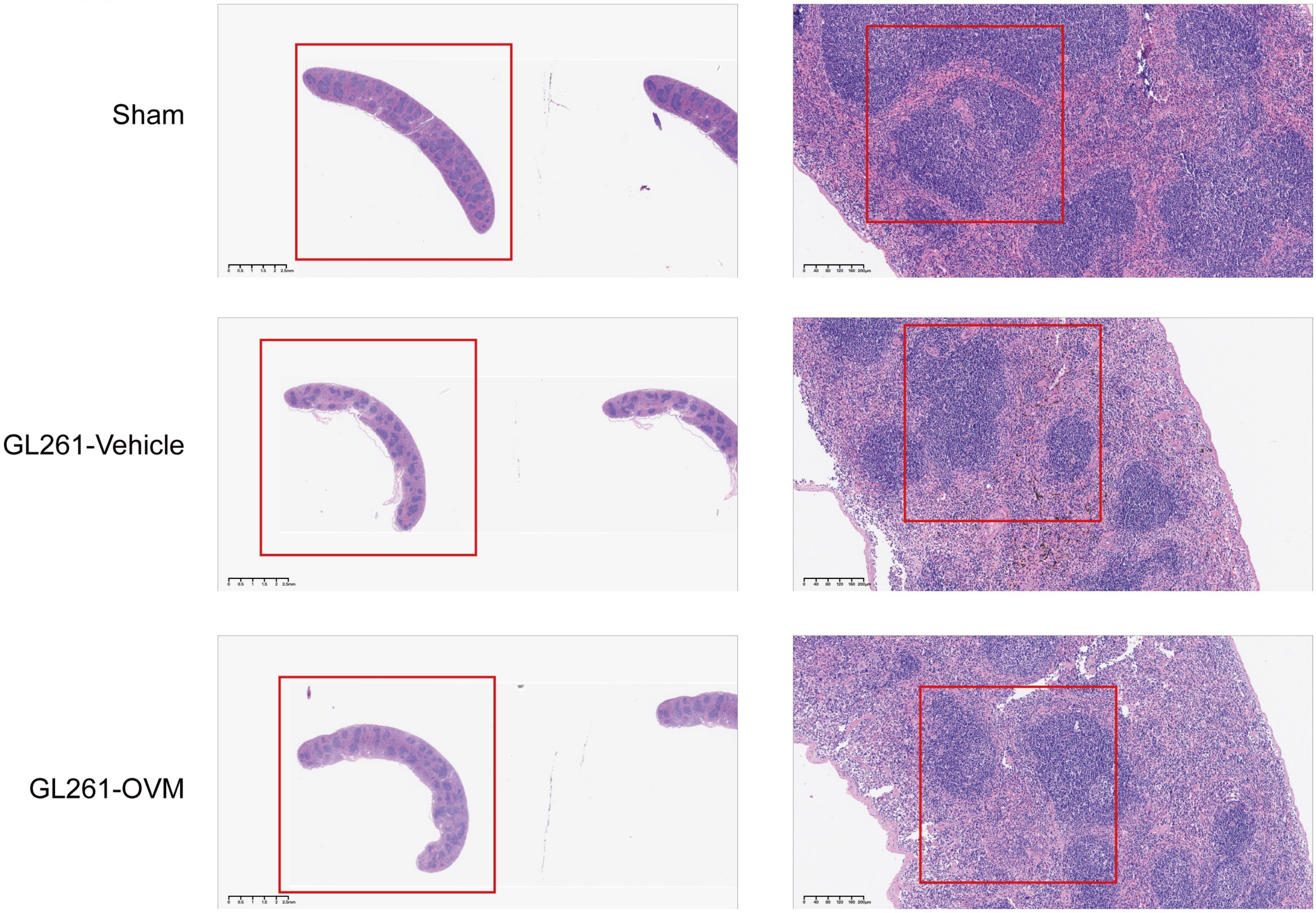

Supplementary Fig. S2H

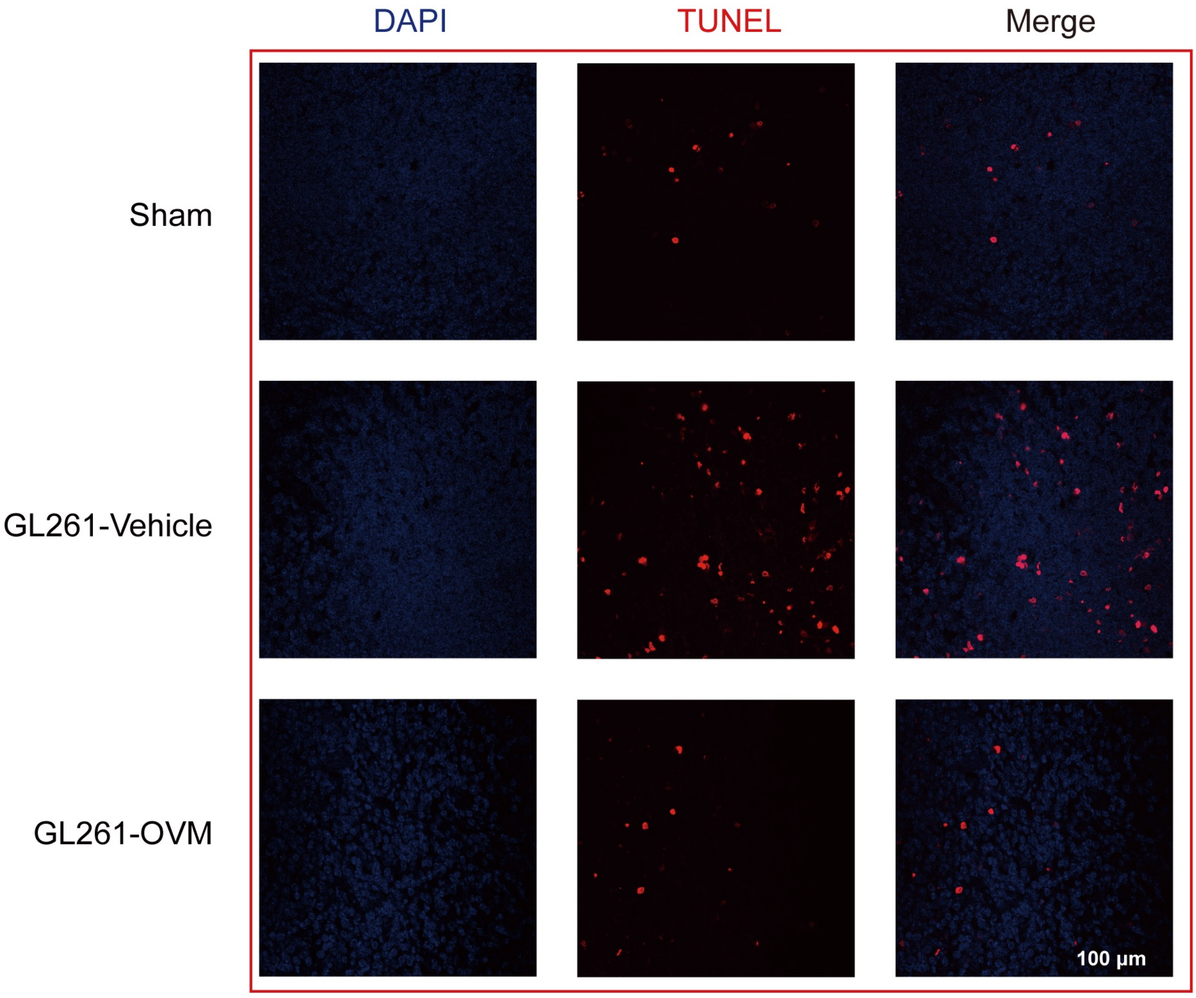

Note: All images in Supplementary Fig. S2H are unprocessed.

Supplementary Fig. S4A

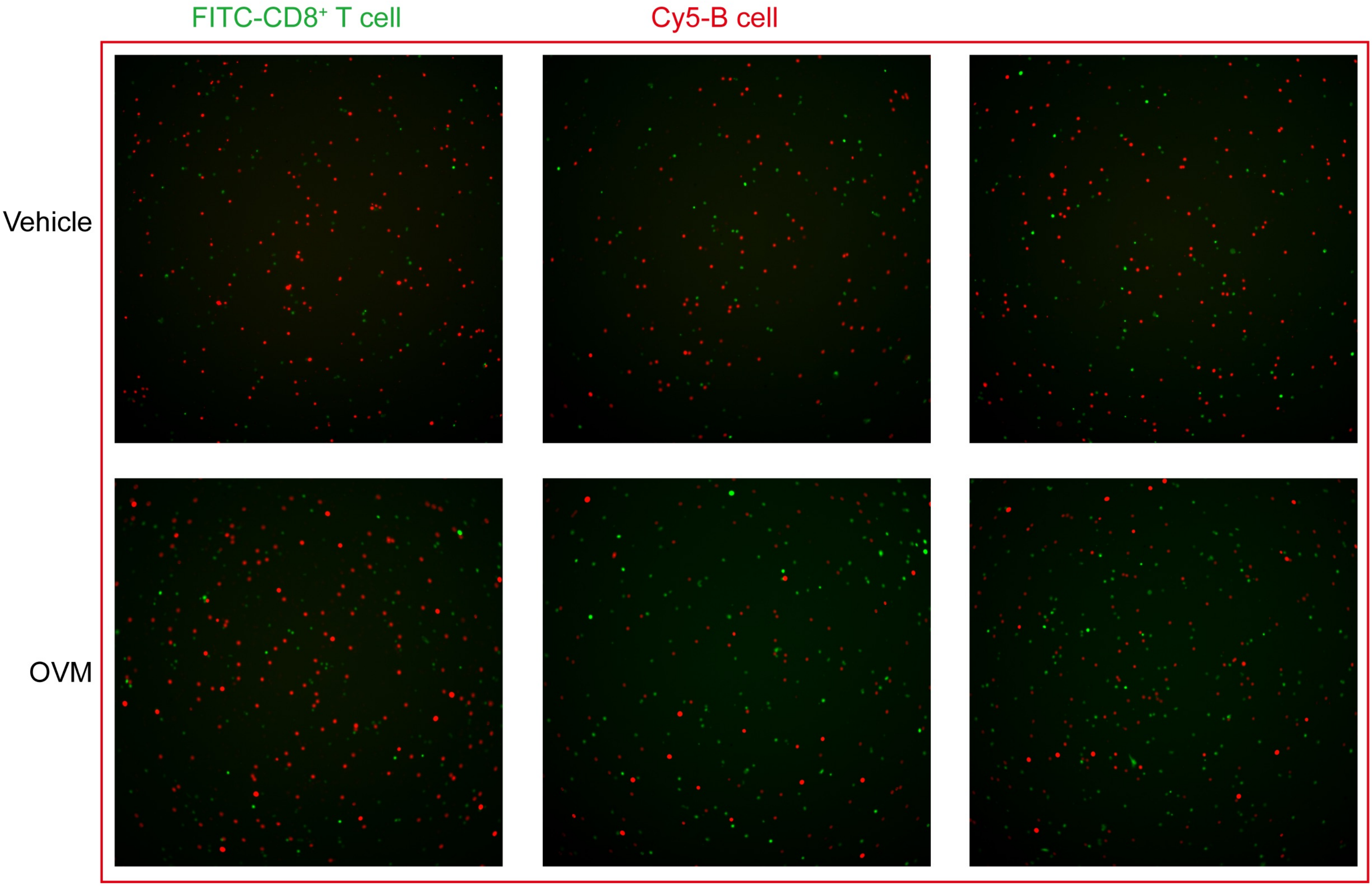

Supplementary Fig. S6A

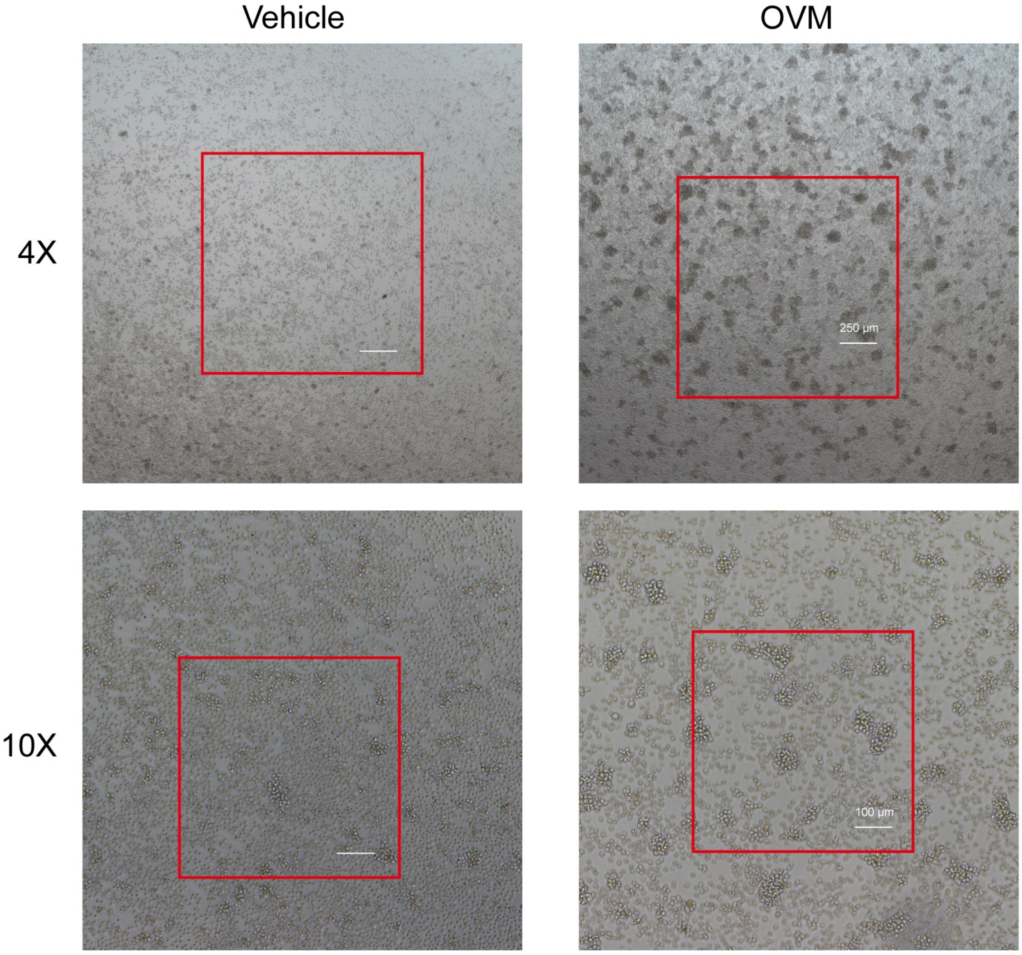

Supplement: Supplementary file 2 — Revised unprocessed images [file 41423_2026_1396_MOESM2_ESM.pdf]
